# Brønsted acidic surfactant [HDMM]^+^ [HSO_4_]^−^: a green microreactor assembly for stereoselective synthesis of novel thiazolyl-pyrazole-chromen-2-ones in water†

**DOI:** 10.1039/d5ra00894h

**Published:** 2025-04-28

**Authors:** Mayuri V. Patil, Pradeep M. Mhaldar, Vrushali M. Mahadik, Rinku Ghanta, Madhulata Shukla, Suraj A. Sonawane, Suresh K. Ghotekar, Gajanan S. Rashinkar, Dattaprasad M. Pore

**Affiliations:** a Department of Chemistry, Shivaji University Kolhapur-416004 Maharashtra India; b Shrimant Bhaiyyasaheb Rajemane Mahavidyalaya Mhaswad Tal; Man, Dist Satara 415509 India; c Diamond Harbour Women's University Sarisha, South 24 Parganas (S) West Bengal 743368 India; d Gram Bharti College Ramgarh, Veer Kunwar Singh University Kaimur Bihar 821110 India; e Rajaram Mahavidyalaya Kolhapur-416004 Maharashtra India; f Centre for Herbal Pharmacology and Environmental Sustainability, Chettinad Hospital and Research Institute, Chettinad Academy of Research and Education Kelambakkam Tamil Nadu 603103 India p_dattaprasad@rediffmail.com

## Abstract

A novel Brønsted acidic surfactant was synthesized and employed as a catalyst for a one-pot multi-component reaction. Small angle X-ray scattering (SAXS) analysis was performed, which confirmed that the micelles exhibited an average diameter of 3.1 nm and average inter-micellar distance of 0.49 nm. Ground state density functional theory (DFT) calculation was performed on the surfactant molecule to optimize the geometrical structure. A series of novel thiazolyl-pyrazole-chromen-2-one derivatives were efficiently synthesized through a convenient one-pot multi-component reaction of substituted 3-acetoacetyl coumarins, thiosemicarbazide and dialkyl acetylene dicarboxylates in water using a novel hexadecyl methyl morpholinium hydrogen sulfate [HDMM]^+^ [HSO_4_]^−^ as surfactant. Operational simplicity, stereoselective synthesis, quick access to the desired products, high purity and good to excellent yields are the key advantages of this approach. This work remarkably highlights the dual novelty as a new class of thiazolyl-pyrazole-chromen-2-one derivatives as well as a [HDMM]^+^ [HSO_4_]^−^ surfactant.

## Abstract 2

A novel Brønsted acidic surfactant, [HDMM]^+^ [HSO_4_]^−^ catalysed one-pot multi-component synthesis of thiazolyl-pyrazole-chromen-2-ones in water.

## Graphical abstract

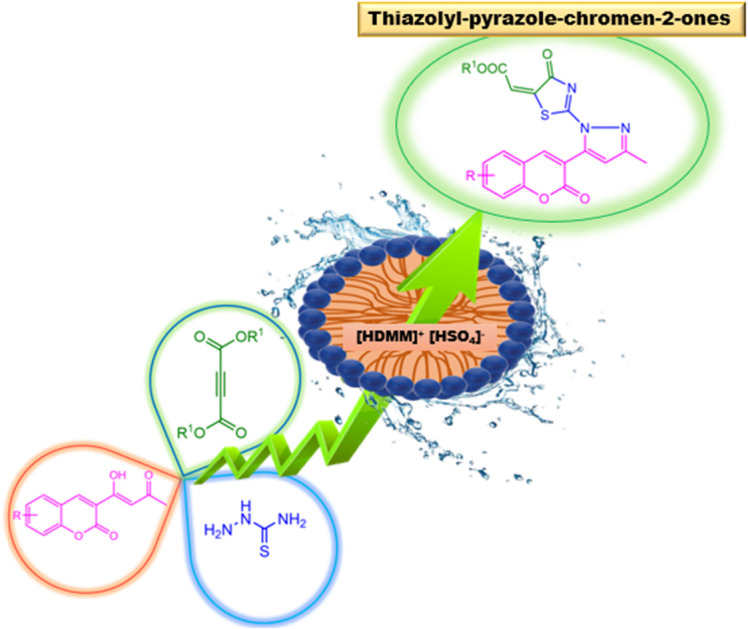

## 1 Introduction

In recent years, the development of sustainable reaction processes is a topic of great interest.^1^ The effort for this began with the use of water as a green solvent in organic transformation.^2–9^ Water is an abundant renewable resource and avoids the production of environmentally harmful waste and high process costs. Additionally, on many occasions, it activates the functional groups by forming hydrogen bonds.^10^ Poor solubility of the reactants is one of the limitations while using aqueous media. Hence, ‘on water’ approach is the most suitable instead of using polar aprotic solvent. The ‘on water’ system involves a surfactant that induces the reaction through micelle in an aqueous medium. The presence of surfactant significantly improves the solubility of hydrophobic compounds in water.

The construction of bioactive scaffolds is known to be the focus of research in organic synthesis. Designing new drugs from hybrid molecules using different pharmacophores may offer remarkable biological activities. Sulfur–nitrogen containing heterocyclic compounds, specifically thiazoles and their derivatives, are a medicinally and pharmaceutically important class of heterocycles. Thiazolidin-4-ones are important moieties in synthetic reactions and exhibit various biological activities, including anticancer,^11^ anti-inflammatory,^12^ antimicrobial,^13^ anticonvulsant,^14^ antifungal,^15^ antitubercular,^16^ anti-HIV,^17^ analgesics,^18^ antimalarial,^19^ HIV inhibitory activity,^20,21^ and hyperglycemic^22^ reversal activity.^23^

**Fig. 1:**
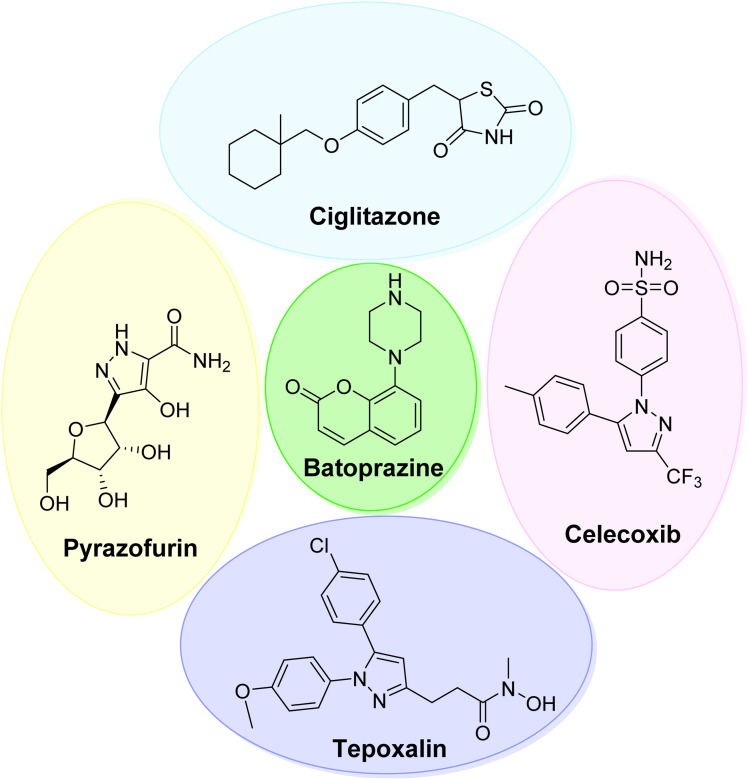
Representative examples of bioactive compounds containing coumarin, pyrazole and thiazolidinone scaffolds.

Conversely, pyrazole and its derivatives exhibit various therapeutic activities^24,25^ such as anti-inflammatory,^26^ antihypertensive,^27^ antimicrobial,^28^ antidiabetic,^29^ and anticancer^30^ activities. Celecoxib and pyrazofurin, which are known as blockbuster drugs, incorporate pyrazole rings into their core structures (Fig. 1). Additionally, compounds of this class play a vital role in organic syntheses.^31,32^

As far as various classes of heterocycles are concerned, coumarins are eminent owing to their excellent pharmacological properties. Coumarins, owing to their structural diversity, are considered an efficient candidate in pharmaceutical chemistry and exhibit a range of biological activities.^33–35^ Anisucoumaramide and clauhainanin-A isolated from *Clausena anisum-olens* and *Clausena hainanensis* exhibit remarkable pharmacological properties.^36,37^ Coumarin derivatives obtained from natural sources include dicoumarol, warfarin, acenocoumarol, and coumachlor. They are widely used to decrease blood coagulation.^38^ In addition, various molecules containing a coumarin skeleton have applications as photosensitizers,^39^ fluorescent chemosensors^40^ for light energy harvesting,^41^ and electroluminescent materials^42^ and in soaps, perfumes, and detergents.^43^

**Scheme 1:**
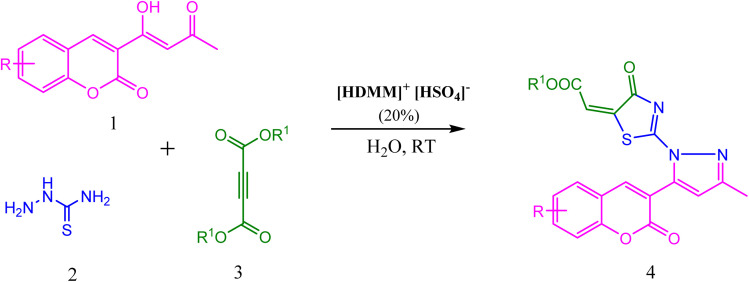
Synthesis of thiazolyl-pyrazole-chromen-2-ones.

Considering the importance of thiazolidine-4-one, pyrazole, and coumarin derivatives, and as a part of our endeavour towards the synthesis of a new class of biologically potent heterocyclic hybrids using a green chemistry protocol, herein, we report a highly efficient method for the diversity-oriented synthesis of thiazolyl-pyrazole-chromen-2-ones *via* a one-pot reaction of acetoacetyl coumarin, thiosemicarbazide, and dialkyl acetylene dicarboxylate in the presence of hexadecyl methyl morpholinium hydrogen sulphate ([HDMM]^+^ [HSO_4_]^−^) as an efficient and reusable Brønsted acidic surfactant catalyst (Scheme 1).

## 2 Results and discussion

**Fig. 2:**
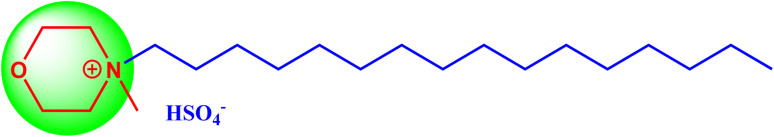
Brønsted acid surfactant catalyst, [HDMM]^+^ [HSO_4_]^−^.

The catalyst significantly affects various factors in organic synthesis; hence, it is a key parameter. The scientific community is continuously making efforts to develop organic transformations in aqueous medium using a benign catalytic system and avoiding toxic catalysts. Hence, initial attention was focused on the design and synthesis of a novel catalyst, *viz* Brønsted acidic surfactant (Fig. 2).

**Scheme 2:**
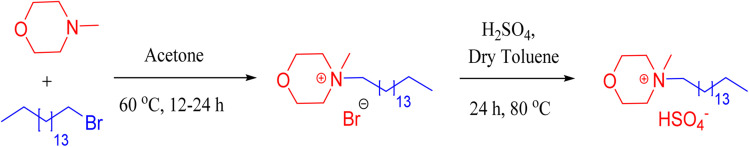
Synthesis of a novel Brønsted acidic surfactant, [HDMM]^+^ [HSO_4_]^−^.

The synthesis of 4-hexadecyl-4-methylmorpholin-4-ium hydrogen sulphate, [HDMM]^+^ [HSO_4_]^−^, is depicted in Scheme 2. The quaternization of 4-methylmorpholine with 1-bromo hexadecane in acetone is carried out by refluxing the reaction mixture at 60 °C for 24 h to afford 4-hexadecyl-4-methylmorpholin-4-ium bromide, [HDMM]^+^ [Br]^−^, followed by anion exchange with conc. H_2_SO_4_ in dry toluene at 80 °C for 24 h furnished 4-hexadecyl-4-methylmorpholin-4-ium hydrogen sulphate, [HDMM]^+^ [HSO_4_]^−^, a Brønsted acidic surfactant. The absence of bromide ions was examined by testing the reaction of the surfactant with AgNO_3_. The synthesized catalyst was confirmed by FTIR, ^1^H, ^13^C NMR, CMC, and TGA analyses. The obtained spectroscopic data fully agreed with the structure of the surfactant catalyst.

**Fig. 3:**
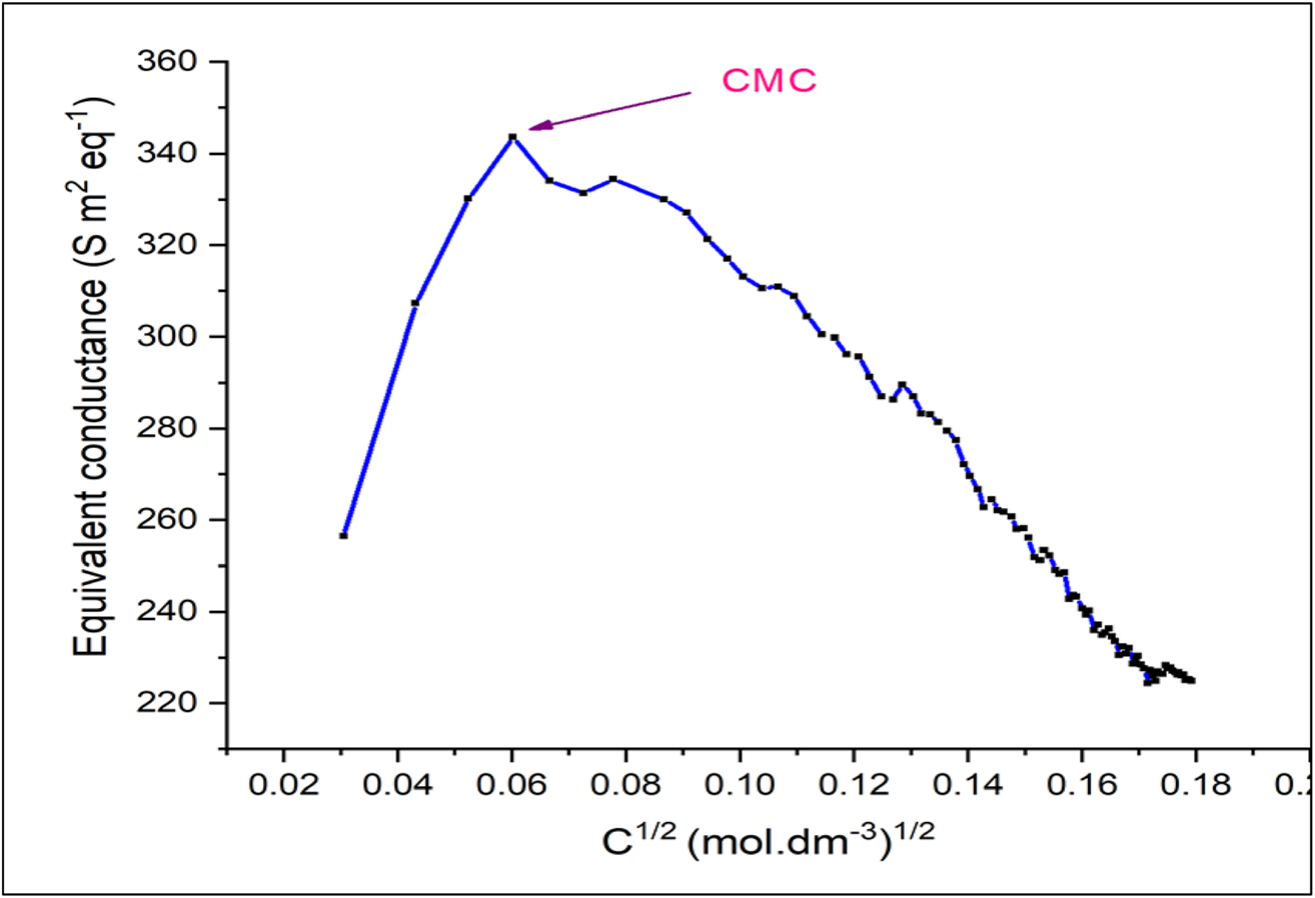
Equivalent conductance as a function of concentration.

The critical micelle concentration (CMC) of a surfactant solution was determined by applying the conductometric method. Fig. 3 illustrates the plot of CMC with coordinates equivalent conductance (*k*) *versus* surfactant concentration. The CMC of [HDMM]^+^ [HSO_4_]^−^ was found to be 0.0037 mol dm^−3^.

**Fig. 4:**
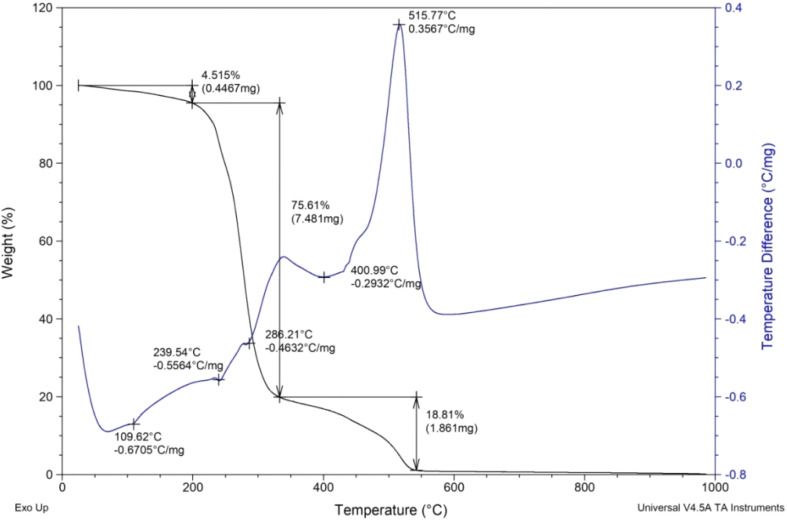
TGA/DTA plot of [HDMM]^+^ [HSO_4_]^−^.

The thermo gravimetric analysis (TGA) was performed at temperature ranging from 25 to 1000 °C under aerobic conditions at 10 °C min^−1^ (Fig. 4). Initially, a weight loss of 4.515% was observed in the temperature range of 25–200 °C owing to the loss of physically adsorbed water from the catalyst. Further, the large weight loss of 75.61% in the range of 200–330 °C is attributed to the exothermic decomposition of the organic moiety. The third weight loss of 18.81% is due to the decomposition of residual carbonaceous species.

### 2.1 Small-angle X-ray scattering (SAXS) analysis

To elucidate the micelle diameter and the intermicellar spacing of [HDMM]^+^ [HSO_4_]^−^, small-angle X-ray scattering (SAXS) analysis was performed. SAXS data for the samples were obtained using a Xenocs SA-France instrument, employing CuKα X-rays with a wavelength of 1.54 Å. The sample was placed in a quartz capillary, and measurements were collected for ten minutes using line collimation. The sample-to-detector distance was 2500 millimeters. The detectors used were an Eiger R 1 M equipped with a vacuum lining and a high-resolution hybrid pixel photon counting system featuring a pixel size of 75 μm. The sample was subjected to 15-minute exposure intervals, during which scattered waves were recorded across a momentum transfer range of *q* = 0.04 to 4 nm^−1^ (eqn (1)):1
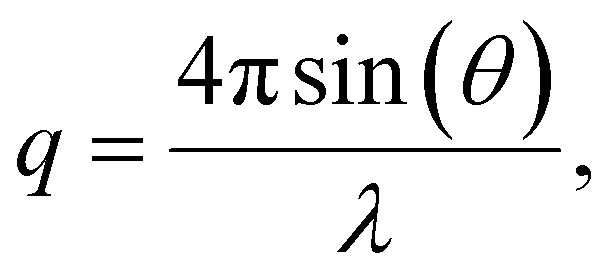
where *λ* = wavelength.

**Fig. 5:**
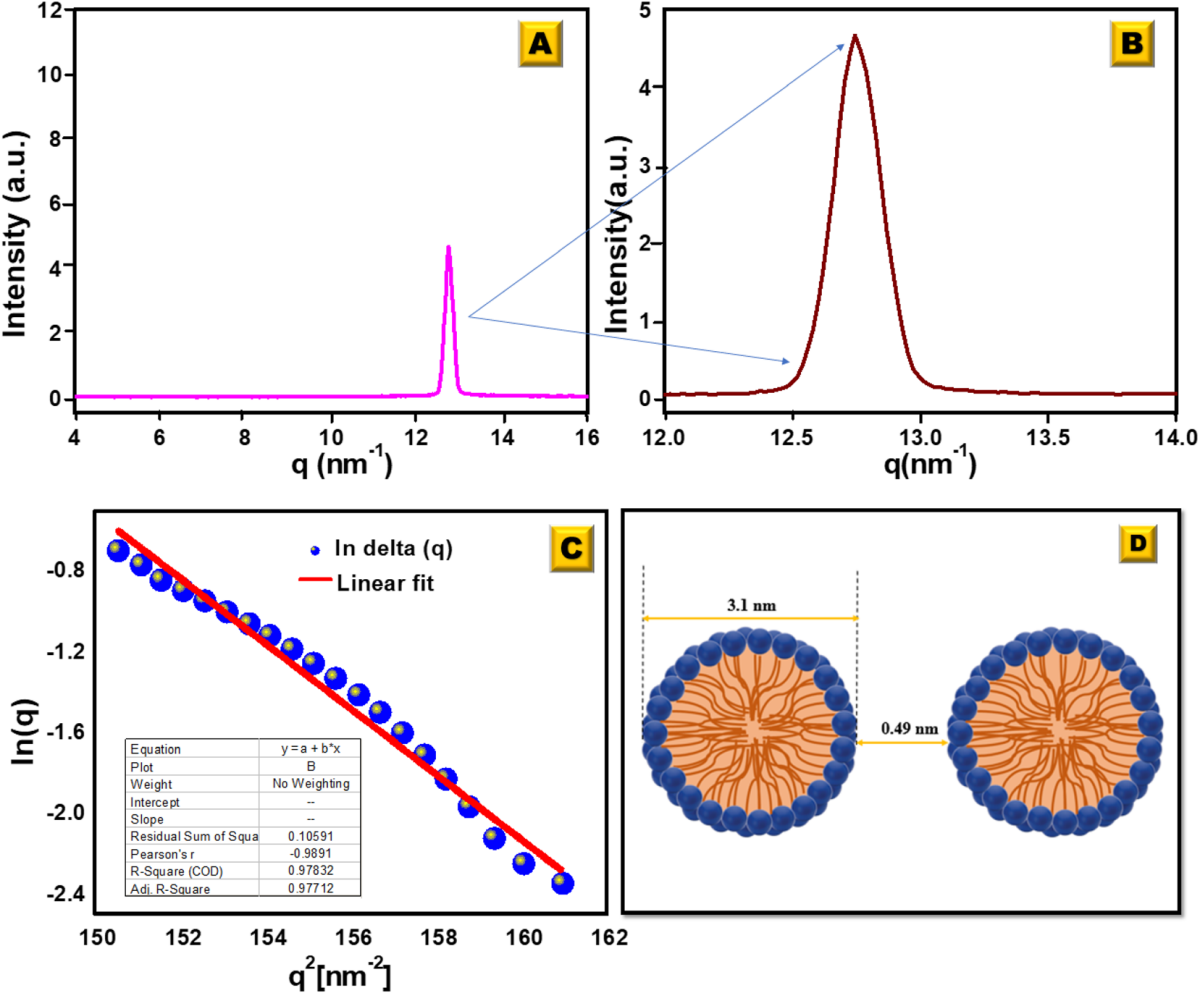
(A) SAXS analysis for the [HDMM]^+^ [HSO_4_]^−^ surfactant in water. (B) Scattered peak position. (C) Guinier's plot. (D) Spacing between the micelle and diameter of the micelle.

Fig. 5a and b show the intensity (*I*) *vs.* scattering vector (*q*) plots obtained from the scattering data of the SAXS measurements of [HDMM]^+^ [HSO_4_]^−^ surfactant micelle assembly in water. X-ray scattering was observed at *q* = 12.74 nm^−1^. The intermicellar separation distance (*d*_Bragg_) was determined to be 0.49 nm (eqn (2)):2
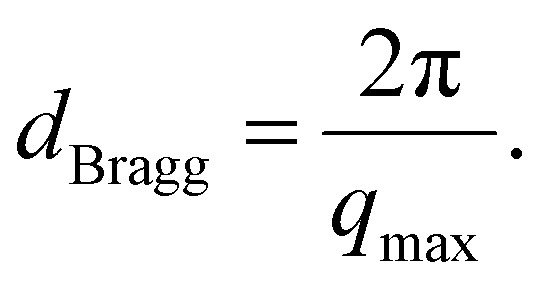


To determine the size of the spherical micelle system, the radius of gyration (*R*_G_) was calculated using Guinier's plot (Fig. 5c). Guinier's plot is obtained from the equation given below:3
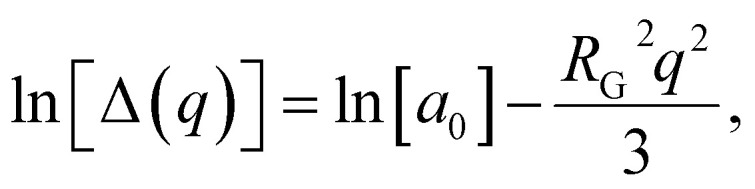
where *a*_0_ is the zero angle intensity.

The radius of the gyration (*R*_G_) was 0.69 nm evaluated from the slope of ln[Δ(*q*)] *vs. q*^2^ plot. The straight line of the data points in the Guinier plot indicates the uniform size of the micelles formed in water. This confirms the introduction of a homogeneous distribution of equal-sized micelles. The average radius of the spherical particle (*R*) was derived from the radius of gyration (*R*_G_) using the equation 
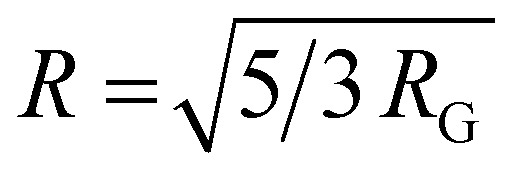
. The average radius of the particle was detected to be 1.55 nm and the diameter is 3.1 nm. The average distance of separation was found to be 0.49 nm.^44^

### 2.2 Molecular geometry optimization

**Fig. 6:**
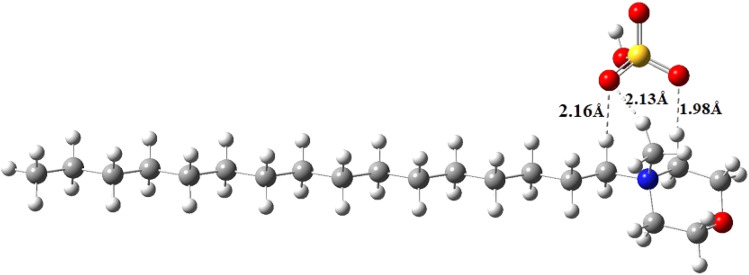
Optimized structure of the surfactant molecule [HDMM]^+^ [HSO_4_]^−^.

**Fig. 7:**
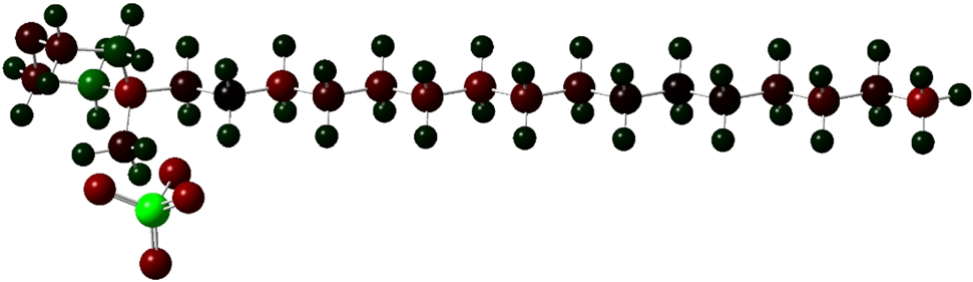
Mulliken charge distribution on the surfactant molecule [HDMM]^+^ [HSO_4_]^−^ (the green color represents the positively charged atom, while the brown color represents the negatively charged atoms).

Ground state Density Functional Theory (DFT) calculation was performed on the surfactant molecule to optimize the geometrical structure. The DFT calculation was carried out at Becke's three-parameter functional and Lee–Yang–Parr hybrid functional (B3LYP) levels using the Gaussian 16 program. The 6-31G++(d,p) basis set was used for geometry optimization.^45,46^ Calculations were performed in the gaseous phase. The optimized structure of the surfactant molecule is shown in Fig. 6. The optimized structure depicts weak hydrogen bonding of lengths 2.16 Å, 2.13 Å, and 1.98 Å existing between the oxygen of the anion and the hydrogen of the cation moiety. Hydrogen bonding is shown by dotted lines in Fig. 6. The Mulliken charge distribution of the optimized molecule calculated at the same level of calculation is shown in Fig. 7.

**Fig. 8:**
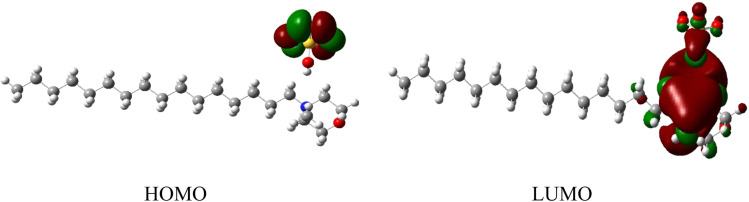
Frontier molecular orbital images of HOMO and LUMO of the surfactant molecule.

The highest occupied molecular orbital (HOMO) and lowest unoccupied molecular orbital (LUMO) of a surfactant molecule are shown in Fig. 8. HOMO is located on the non-bonding orbital of the oxygen atom of the anion moiety, while LUMO is localized mainly on the cyclohexane ring of the cation moiety.

### 2.3 Optimization study

After these major achievements, attention was turned towards exploring the catalytic efficiency of the synthesized catalyst, [HDMM]^+^ [HSO_4_]^−^, to synthesize thiazolyl-pyrazole-chromen-2-one derivatives. The optimization of the catalyst was carried out using the model reaction of acetoacetyl coumarin, thiosemicarbazide and dimethyl acetylene dicarboxylate (DMAD) at room temperature. No significant yield was obtained for the reaction without a catalyst (Table 1, entry 1). Therefore, K_2_CO_3_, NH_2_–SO_3_H, *p*-TSA, l-proline, cetyltrimethyl ammonium bromide (CTAB), sodium dodecyl sulphate (SDS), Triton X-100, sodium dioctyl sulfosuccinate (SDOSS), benzethonium chloride [BZT]^+^ Cl^−^, [BZT]^+^ AlCl_4_^−^ and [HDMM]^+^ [HSO_4_]^−^ are screened for model reaction (Table 1, entries 2–12).

**Table 1:** Screening of catalysts for the formation of thiazolyl-pyrazole-chromen-2-ones (4a) a

Entry	Catalyst	Catalyst load (mol%)	Time (h)	Yield^b^ (%)
1	—	—	12–24	20
2	K_2_CO_3_	20	20	40
3	NH_2_–SO_3_H	20	6.2	78
4	*p*-TSA	20	5	80
5	l-Proline	20	7	70
6	CTAB	20	18	55
7	SDS	15	24	58
8	Triton X 100	20	24	61
9	SDOSS	20	20	48
10	[BZT]^+^ Cl^−^	20	11	55
11	[BZT]^+^ AlCl_4_^−^	20	6	75
12	[HDMM]^+^ HSO_4_^−^	10	4	80
13	[HDMM]^+^ HSO_4_^−^	15	3	83
14	[HDMM]^+^ HSO_4_^−^	20	2.5	89
15	[HDMM]^+^ HSO_4_^−^	25	3	90

a Reaction conditions: acetoacetyl coumarin (1 mmol), thiosemicarbazide (1 mmol), dialkyl acetylene dicarboxylate (1 mmol), specific catalyst, water (5 mL), RT.

b Isolated yield.

K_2_CO_3_ as a catalyst provides a low yield of the desired product (Table 1, entry 2). The yield was sufficiently increased for the reactions in the acid catalysts (Table 1, entries 3–5, and 11). Commercially available surfactants, *viz* cetyltrimethyl ammonium bromide (CTAB), sodium dodecyl sulfate (SDS), Triton X-100, sodium dioctyl sulfosuccinate (SDOSS) and benzethonium chloride [BZT]^+^ Cl^−^, gave moderate yield in aqueous medium (Table 1, entries 6–10). Pleasingly, [HDMM]^+^ [HSO_4_]^−^ exhibited a high yield (Table 1, entry 14). Thus, amongst the catalysts screened, [HDMM]^+^ [HSO_4_]^−^ was found to be superior for performing the reaction at room temperature.

The effect of catalyst loading was also studied for the model reaction. The 20 mol% of [HDMM]^+^ [HSO_4_]^−^ was found to be tolerable in promoting this reaction (Table 1, entries 12–15). Catalyst loading greater than 20% did not positively influence both the yield and reaction time.

**Fig. 9:**
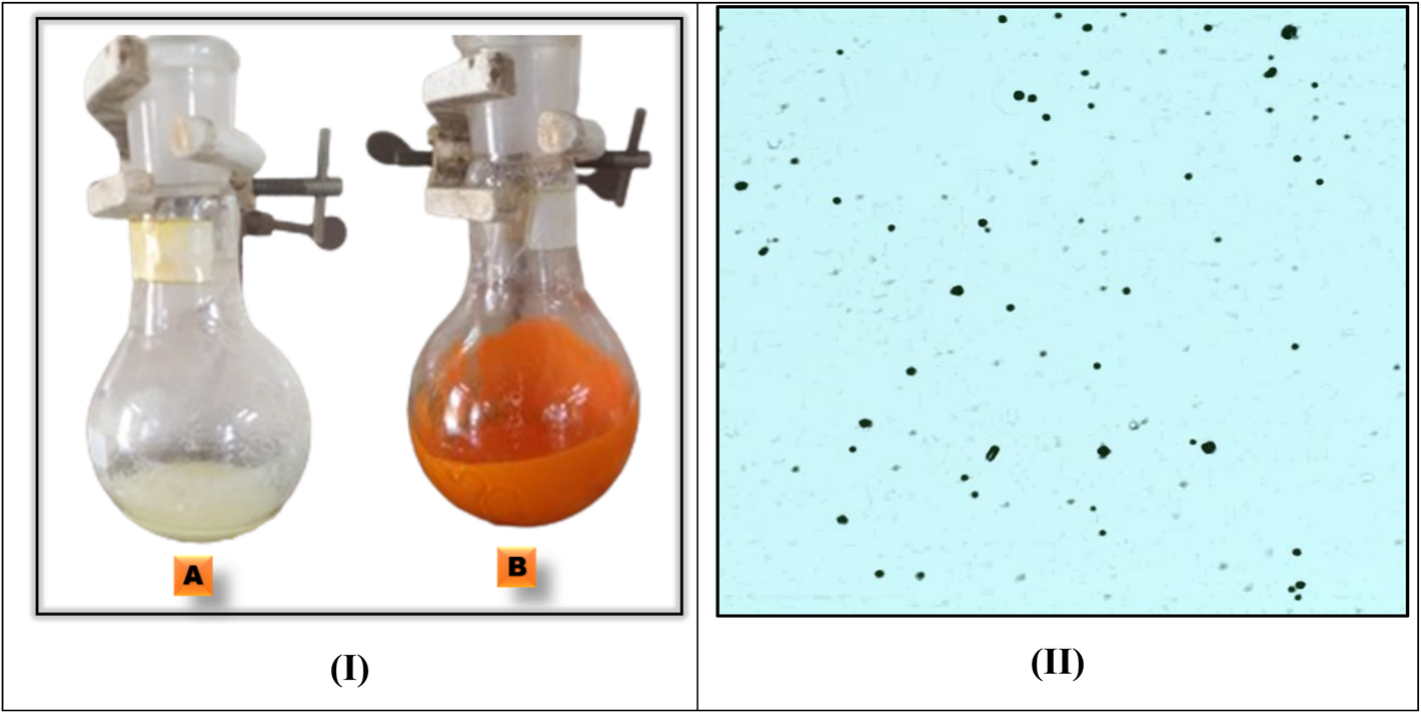
(I) (A) Reaction mixture at intermediate (acetoacetylcoumarine + thiosemicarbazide). (B) Reaction mixture after the completion of the reaction (after the addition of DMAD). (II) Optical micrograph image of the reaction mixture in water.

The reaction mixture of acetoacetylcoumarin and thiosemicarbazide was converted into a homogeneous solution by adding a surfactant. The formation of white turbid emulsion confirms the formation of micelles or colloidal aggregates [Fig. 9(I)]. Finally, an orange precipitate was obtained after the addition of DMAD, indicating the completion of the reaction. The formation of spherical emulsion droplets (microbubbles) in the aqueous medium was confirmed by taking an optical microscopic image [Fig. 9(II)].

Notably, the workup of the reaction was carried out by simple filtration and recrystallization in hot ethanol to produce an extremely pure product. Based on spectral information, the structure of the product was confirmed (4a). Infrared analysis of compound 4a exhibited absorption bands at 1719, 1687 and 1347 cm^−1^ due to lactone (C=O), imine (C=N), and (C–S) stretching frequencies, respectively. The ^1^H NMR and ^13^C NMR spectra of 4a concluded that the product was isometrically pure. The ^1^H NMR study of methyl (*E*)-2-(2-(3-methyl-5-(2-oxo-2*H*-chromen-3-yl)-1*H*-pyrazol-1-yl)-4-oxothiazol-5(4*H*)ylidene) acetate 4a showed that the singlet of vinylic proton at *δ* = 6.73 ppm clearly indicates the *E*-configuration of the exocyclic double bond of the thiazolidinone ring.^47–49^ The presence of vinylic proton above 6.90 ppm supported the *Z*-configuration.^50^ The peaks at *δ* 2.34 ppm and 3.87 ppm are due to methyl and methoxy protons, respectively. The singlet corresponding to the methine proton of pyrazole and coumarin is observed at *δ* 6.69 and 8.41 ppm, respectively. The aromatic protons of the coumarin skeleton were observed at *δ* 7.37–8.02 ppm. ^13^C NMR analysis also confirmed the structural identity, with resonance observed at *δ* 15.89 (–CH_3_), 52.06 (–OMe), 100.42 (pyrazol–CH), 114.81, 116.15, 118.59, 118.77, 124.82, 126.10, 129.20 (coumarin Ar-CH), 129.79, 132.29, 140.71 (pyrazole C–N), 141.77 (pyrazole C=N), 142.72, 146.05 (coumarin –CH), 153.06 (=CH–COOMe), 153.24 C=C thiazole), 161.09 (C=N thiazole), 163.91 (–CO lactone), 166.12 (–CO ester) and 170.89 (–CO–amide). The molecular ion peak of compound 4a was found in the mass spectrum at *m*/*z* = 395.39 [M]^+^, corresponding to the molecular formula C_19_H_13_N_3_O_5_S. All the above spectroscopic data clearly indicate the formation of the target product.

With improved reaction conditions in hand, we expanded the scope of the reaction using various structurally diverse acetoacetyl coumarin derivatives with dimethyl and diethyl acetylene dicarboxylates (DMAD and DEAD) [Table 2]. Interestingly, acetoacetyl coumarin with electron-withdrawing and electron-donating substituents was almost inevitably transformed into its respective targets with an excellent yield (Table 2, product 4c–4i).

**Table 2:** Synthesis of a combinatorial library of thiazolyl-pyrazole-chromen-2-one derivatives^a^

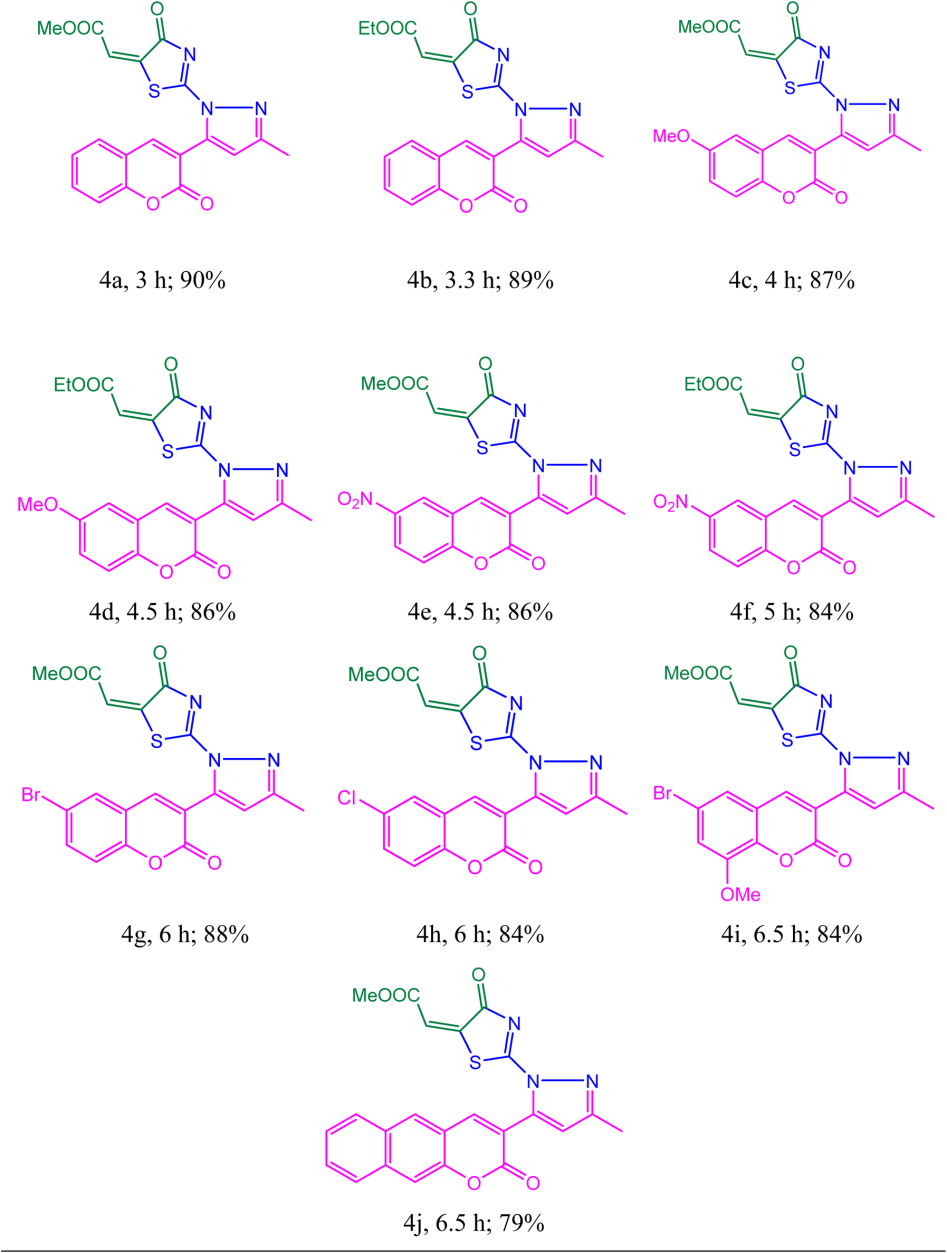

a Reaction conditions: acetoacetylcoumarin (1 mmol), thiosemicarbazide (1 mmol), dialkyl acetylene dicarboxylate (1 mmol), catalyst: [HDMM]^+^ [HSO_4_]^−^ (20%), water (5 mL), room temperature.

Eventually, the competency of the reaction was examined using an acetoacetyl coumarin derivative synthesized from 2-hydroxy naphthaldehyde, and it was found that the reaction performed well with a good yield (Table 2, product 4j).

**Scheme 3:**
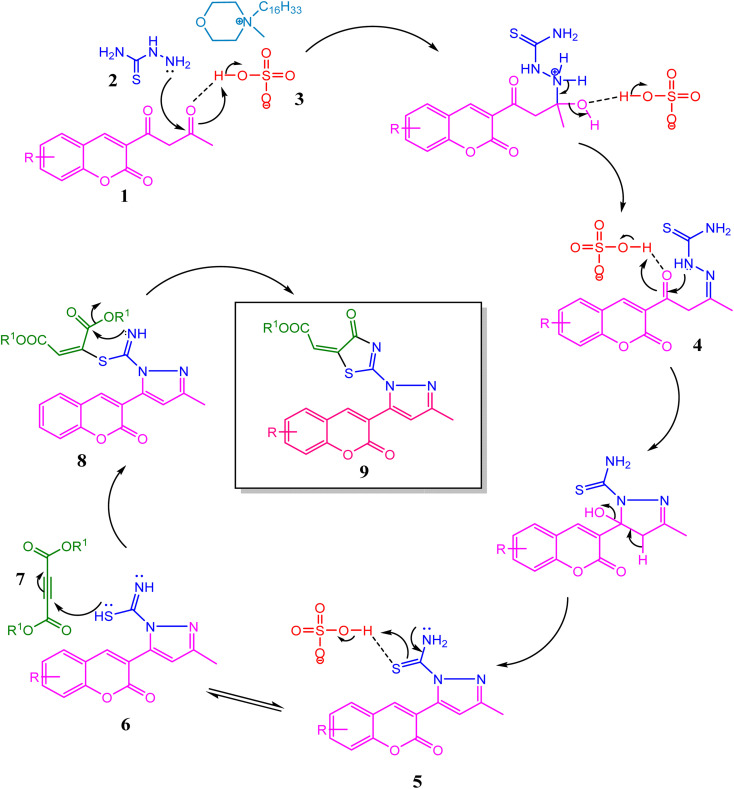
Plausible reaction mechanism for the synthesis of thiazolyl-pyrazole-chromen-2-ones.

A plausible mechanism for the formation of thiazolyl-pyrazole-chromen-2-ones is depicted in Scheme 3. Initially, the condensation of thiosemicarbazide 2 with acetoacetyl coumarin 1 results in the formation of Knorr-pyrazole skeleton 5. The sulphur atom of 6 as thiol after prototropic tautomeric shift attacks one of the ethynyl carbons of DMAD 7 in a Michael addition manner to yield *S*-alkylated intermediate 8, followed by an intramolecular amidation reaction to yield product 9. Overall, the reaction generates one C–S, one C=N, and two C–N bonds. Simultaneously, thiazole and pyrazole heterocycles were developed successively.

**Fig. 10:**
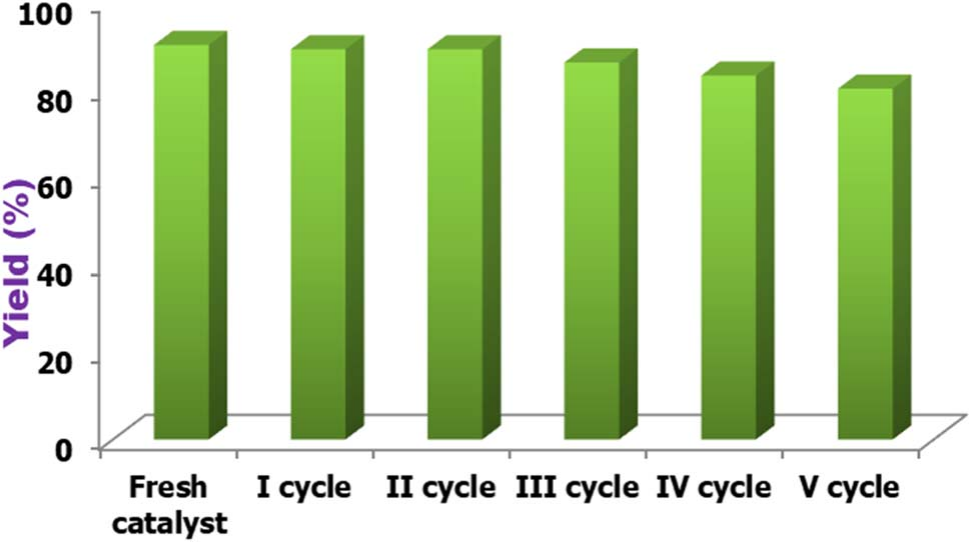
Recyclability study of [HDMM]^+^ [HSO_4_]^−^.

Because catalyst reusability is an essential economic consideration, recovery and reusability experiments were conducted for the reaction of acetoacetyl coumarin, thiosemicarbazide, and dimethyl acetylene dicarboxylate. Following the reaction, the product was filtered and washed multiple times with 25 mL of water. The collected filtrate was concentrated on a rotary evaporator to 5 mL, and the filtrate remaining in the flask was washed with diethyl ether before being reused immediately by adding substrates in the next cycle, with no additional purification. As shown in Fig. 10, the catalyst can be reused five times without a significant decrease in catalytic activity.

## 3 Conclusion

In conclusion, we developed a new, facile, one-pot multi-component synthesis of substituted thiazolyl-pyrazole-chromen-2-ones from acetoacetyl coumarin, thiosemicarbazide, and dialkyl acetylene dicarboxylate in the presence of [HDMM]^+^ [HSO_4_]^−^ as an effective Brønsted acidic surfactant. The small angle X-ray scattering (SAXS) analysis and ground state Density Functional Theory (DFT) calculation confirmed the size and geometrical structure of the surfactant molecule. This approach provides various advantages, such as the use of water as a universal solvent, short reaction time, good yields, wide substrate scope, easy work-up, and furnishing a pure product without tedious column chromatography. This transformation established the formation of four bonds: one C–S, one C=N, and two C–N bonds. It also generated thiazole and pyrazole heterocycles. The synthesized derivatives containing three heterocyclic rings may be beneficial in drug discovery.

## Data availability

The data supporting this article have been included as part of the ESI.†

## Author contributions

Mayuri Patil: conceptualization, methodology, writing – original draft preparation. Pradeep Mhaldar: methodology, software, data curation, Vrushali Mahadik: visualization, writing – reviewing and editing Rinku Ghanta and Madhulata Shukla: software, validation. Suraj Sonawane: writing – reviewing and editing Suresh K. Ghotekar: visualization, writing – reviewing and editing Gajanan Rashinkar: supervision, writing original draft Dattaprasad Pore: supervision, visualization, writing – reviewing and editing.

## Conflicts of interest

There are no conflicts to declare.

## Supplementary Material

RA-015-D5RA00894H-s001
