# Supplementary material for: Brønsted acidic surfactant [HDMM]+ [HSO4]−: a green microreactor assembly for stereoselective synthesis of novel thiazolyl-pyrazole-chromen-2-ones in water
Source: RSC Adv. 2025 Apr 28;15(18):13753–62. doi: 10.1039/d5ra00894h (PMC12036754; doi:10.1039/d5ra00894h)
Supplement: RA-015-D5RA00894H-s001 [file RA-015-D5RA00894H-s001.pdf]

Supplementary Information (SI) for RSC Advances.
This journal is © The Royal Society of Chemistry 2025

Supporting information

Bronsted acidic surfactant [HDMM]$^+$ [HSO_4]$^-$: A Green microreactor assembly for stereoselective synthesis of novel thiazolyl-pyrazole-chromen-2-one in water

Mayuri V. Patil[a], Pradeep M. Mhaldar[b], Vrushali M. Mahadik[a], Rinku Ghanta[c], Madhulata Shukla[d] Suraj A. Sonawane[e], Suresh K. Ghotekar[f], Gajanan S. Rashinkar[a], and Dattaprasad M. Pore[a]*

[a]*Department of Chemistry, Shivaji University, Kolhapur- 416004, Maharashtra, India*

[b]*Shrimant Bhaiyyasaheb Rajemane Mahavidyalaya, Mhaswad Tal; Man, Dist : Satara 415509 India*

[c]*Diamond Harbour Women's University, Sarisha, South 24 Parganas (S), West Bengal, 743368 India*

[d] *Gram Bharti College Ramgarh, Veer Kunwar Singh University, Kaimur, Bihar, 821110*

[e] *Rajaram Mahavidyalaya, Kolhapur-416004, Maharashtra, India*

[f] *Centre for Herbal Pharmacology and Environmental Sustainability, Chettinad Hospital and Research Institute, Chettinad Academy of Research and Education, Kelambakkam, Tamil Nadu 603103, India*

**E-mail: p_dattaprasad@rediffmail.com*

	**Table of content**	**Page No.**
A.	General Information	1
B.	General Procedure	1
C.	Spectral data of synthesized surfactant [HDMM]$^+$[HSO4]$^-$ and thiazolyl-pyrazole-chromen-2-one	2-28

A) General:

Various substituted acetoacetyl coumarins were synthesized from salicylaldehyde and 4-hydroxy-6-methyl pyrone. Dialkyl acetylene dicarboxylates (Sigma Aldrich) and thiosemicarbazide (Alfa Aesar) were used as received. Melting points (M.P) were determined in open capillary and are uncorrected. IR spectra were recorded on ATR-IR-4600 spectrometer. NMR spectra were recorded on Bruker AMX-400 spectrometer (400 MHz ^{1}H NMR and 100 MHz for ^{13}C NMR) in DMSO-d_6 employing TMS as an internal standard and δ values are expressed in ppm. HRMS spectra were recorded on JEOL GC MATE-II HR Mass spectrometer from IIT Madras.

B) Typical procedure:

Synthesis of surfactant $[HDMM]^+$ $[HSO4]^-$:

A solution of 4-methyl morpholine (5 g, 0.058 mol) and 1- bromohexadecane (17.69 g, 0.058 mol) in acetone (40 mL) was refluxed at 60 °C for 12-24 h. The white precipitate was filtered after cooling and washed with diethyl ether yielded 4-hexadecyl-4-methyl morpholin-4- ium bromide $[HDMM]^+[Br]^-$ in 96 % yield.

The anion exchange was performed by adding equimolar quantity of sulfuric acid (H_2SO_4) at 80 °C in dry toluene for 24 h and the final product $[HDMM]^+$ $[HSO_4]^-$ was obtained as a waxy solid after separating two phases. The product was washed with ethyl acetate to get pure product.

Synthesis of thiazolyl-pyrazole-chromen-2-one:

In a 50 mL round bottom flask, acetoacetyl coumarin, (1 mmol), thiosemicarbazide (1 mmol) and $[HDMM]^+$ $[HSO_4]^-$ surfactant (20 mol %) in water (5 mL) was stirred at room temperature for 1-2 h to afford chromeno pyrazole carbothioamide intermediate. Then dialkyl acetylene dicarboxylate (1 mmol) was added in the resulting reaction mixture and stirred at room temperature for time specified in **Table 2**. The progress of reaction was monitored by TLC. After completion of the reaction, reaction mixture was filtered and washed with water. The product was recrystallized in ethanol to furnish corresponding pure thiazolyl-pyrazole- chromen-2-one. These synthesized products were characterized by IR, ^{1}H,^{13}C NMR and Mass analysis.

Spectral data of synthesized novel Bronsted acid surfactant catalyst 4-hexadecyl-4-methylmorpholin-4-ium hydrogen sulphate, $[HDMM]^+[HSO4]^-$

Brownish waxy solid; IR **(Fig 1)**: 3411, 2918, 2851, 1686, 1642, 1466, 1365, 1174, 1124, 1042, 889, 760, 720, 653 cm^{-1}; ^1H NMR (400 MHz, $CDCl_3$) **(Fig 2)**: δ 0.86-0.89 (t, 3H), 1.25 (s, 26H), 1.77 (s, 2H), 3.44 (s, 3H), 3.56-3.61 (m, 2H), 3.67-3.76 (m, 4H), 4.01-4.05 (m, 2H), 4.12- 4.17 (m, 2H), 8.47 (br s, 1H); ^{13}C NMR (100MHz, $CDCl_3$) **(Fig 3)**: δ 14.34, 22.17, 22.92, 26.60, 29.58, 29.61, 29.79, 29.87, 29.93, 29.99, 32.17, 47.86, 60.06, 65.71 ppm.

Spectral data of thiazolyl-pyrazole-chromen-2-one derivatives

Entry 4a, Table 2: methyl (*E*)-2-(2-(3-methyl-5-(2-oxo-2*H*-chromen-3-yl)-1*H*-pyrazol-1-yl)-4-oxothiazol-5(4*H*)-ylidene)

Orange solid; Obs. m.p. 280–282 °C. IR **(Fig. 4)**: 3158, 3086, 2899, 2778, 2396, 1725, 1605, 1521, 1444, 1333, 1195, 1118, 1082, 1015, 938, 866, 799, 754, 694, 650, 584 cm-1; 1H NMR (400 MHz, DMSO-d_6) **(Fig. 5)**: δ 2.26 (s, 3H, $-CH_3$), 3.79 (s, 3H, $-OCH_3$), 6.69 (s, 1H, pyrazole-CH), 6.73 (s, 1H, vinyl -CH), 7.37-7.45 (m, Ar-2H), 7.66-7.70 (dd, Ar-1H, J = 7.2 Hz, 1.6 Hz), 7.97-7.99 (d, Ar-1H, J = 7.6 Hz), 8.76 (s, 1H, coumarin -CH) ppm; ^{13}C NMR (100 MHz, DMSO-d_6) **(Fig. 6)**: δ 15.89, 52.50, 100.42, 114.81, 116.15, 118.59, 118.77, 124.81, 126.10, 129.20, 140.70, 141.77, 146.06, 153.05, 153.24, 161.09, 163.91, 166.12, 170.89 ppm; HRMS **(Fig. 7)**: Mass calculated for $[C_{19}H_{13}N_3O_5S]$: 395.39 (M^+) ; Obs. Mass: 395.38 (M^+).

Entry 4b, Table 2 : ethyl (*E*)-2-(2-(3-methyl-5-(2-oxo-2*H*-chromen- 3-yl)-1*H*-pyrazol-1-yl)-4-oxothiazol-5(4*H*)-ylidene)acetate

Orange solid; Obs. m.p. 290 ^{o}C; IR **(Fig. 8)**: 3162, 3079, 1725, 1601, 1441, 1335, 1193, 1122, 1010, 755, 696 cm^{-1}; ^1H NMR, (400 MHz, DMSO-d_6) **(Fig. 9)**: δ 1.27- 1.30 (t,3H), 2.26 (s, 3H, $-CH_3$), 4.24-4.29 (q, 2H, $-OCH_2$), 6.68 (s, 1H, pyrazole -CH), 6.75 (s, 1H, vinyl-CH), 7.36-7.49 (m, Ar-2H), 7.66-7.68 (d, Ar-1H, J = 8.4 Hz), 7.96-7.98 (d, Ar-1H, J = 7.6 Hz), 8.77 (s, 1H, coumarin -CH) ppm; ^{13}C NMR (100 MHz, DMSO-d_6) **(Fig. 10)**: δ 14.02, 17.35, 56.75, 105.35, 113.56, 116.14, 119.71, 122.61, 124.33, 125.39, 128.38,

136.23, 140.96, 142.75, 146.94, 150.80, 153.80, 156.35, 161.83, 167.76, 170.41 ppm; HRMS **(Fig. 11)**: Mass calculated for [$C_{20}H_{15}N_3O_5S$]: 409.42 (M^+) ; Obs. Mass: 409.41 (M^+).

Entry 4c, Table 2: methyl (*E*)-2-(2-(5-(6-methoxy-2-oxo-2*H*-chromen-3-yl)-3-methyl-1*H*-pyrazol-1-yl)-4-oxothiazol-5(4*H*)- ylidene)acetate

Red solid; Obs. m.p. 284 °C; IR **(Fig. 12)**: 3161, 3073, 1713, 1603, 1525, 1505, 1428, 1379, 1332, 1288, 1196, 1122, 1023, 940, 902, 862, 810, 766, 705, 615, 592 cm^{-1} ; ^{1}H NMR (400 MHz, DMSO-d_6) **(Fig. 13)**: δ 2.26 (s, 3H, -CH_3), 3.31 (s, 3H, -OCH_3), 3.81 (s, 3H, -OCH_3), 6.72 (s, 1H, pyrazole - CH), 6.74 (s, 1H, vinyl -CH), 7.25-7.29 (m, Ar-1H), 7.33-7.40 (m, Ar-1H), 7.58-7.58 (d, Ar-1H, *J* = 3.2 Hz), 8.13 (s, 1H, coumarin - CH) ppm ; ^{13}C NMR (100 MHz, DMSO-d_6) **(Fig. 14)**: δ 15.91, 52.54, 56.39, 105.23, 114.83, 117.35, 123.80, 124.35, 125.71, 127.80, 135.38, 140.02, 145.96, 149.33, 150.78, 154.61, 157.33, 161.35, 166.14, 167.76 ppm; HRMS **(Fig. 15)**: Mass calculated for [$C_{20}H_{15}N_3O_6S$] :425.42 (M^+) ; Obs. Mass: 425.41 (M^+).

Entry 4d, Table 2 : ethyl (*E*)-2-(2-(5-(6-methoxy-2-oxo-2*H*- chromen-3-yl)-3-methyl-1*H*-pyrazol-1-yl)-4-oxothiazol-5(4*H*)-ylidene)acetate

Red solid; Obs. m.p. 292 °C; IR **(Fig. 16)**: 3162, 3073, 1707, 1595, 1506, 1430, 1323, 1193, 1122, 1027, 826, 814, 708, 619 cm^{-1}; ^{1}H NMR (400 MHz, DMSO-d_6) **(Fig. 17)**: δ 1.26-1.30 (t, 3H), 2.26 (s, 3H, -CH_3), 3.81 (s, 3H, -OCH_3), 4.23-4.28 (q, 2H, -OCH_2), 6.71 (s, 1H, pyrazole -CH), 6.71 (s, 1H, vinyl -CH), 7.25-7.29 (m, Ar-1H), 7.37- 7.39 (m, Ar-1H), 7.56-7.57 (d, Ar-1H, *J* = 3.2 Hz), 8.72 (s, 1H, coumarin -CH) ppm; ^{13}C NMR (100 MHz, DMSO-d_6) **(Fig. 18)**: δ 14.94, 18.76, 54.91, 58.41, 105.56, 115.35, 116.14, 119.71, 122.61, 124.33, 125.39, 128.38, 136.23, 140.96, 142.75, 146.02, 148.80, 153.80, 158.35, 160.83, 165.35, 168.75 ppm; HRMS **(Fig. 19)**: Mass calculated for [$C_{21}H_{17}N_3O_6S$]: 439.45 (M^+) ; Obs. Mass: 439.43 (M^+).

Entry 4e, Table 2 : methyl (*E*)-2-(2-(3-methyl-5-(6-nitro-2-oxo-2*H*- chromen-3-yl)-1*H*-pyrazol-1-yl)-4-oxothiazol-5(4*H*)-ylidene)acetate

Orange solid; Obs. m.p. 298 °C; IR **(Fig. 20)**: 3101, 3077, 2358, 1836, 1742, 1700, 1617, 1525, 1468, 1335, 1243, 1196, 1101, 1017, 923, 819, 737, 605, 592 cm^{-1}; ^{1}H NMR (400 MHz, DMSO-d_6) **(Fig. 21)**: δ 2.27 (s, 3H, -CH_3), 3.80 (s, 3H, -OCH_3), 6.68 (s, 1H, pyrazole -CH), 6.74 (s, 1H, vinyl -CH), 7.64-7.66 (d, Ar-1H, *J* = 8.8 Hz), 8.43-8.46 (dd, Ar-1H, *J* = 1.6 Hz, 9.2 Hz), 8.75 (s, 1H, coumarin -CH), 8.880- 8.889 (d, Ar-1H, *J* = 3.6 Hz)

ppm; ^{13}C NMR (100 MHz, DMSO-d_6) **(Fig. 22)**: δ 14.89, 53.50, 101.42, 115.81, 116.15, 117.59, 118.77, 125.81, 126.10, 128.20, 140.70, 141.77, 147.06, 153.95, 154.24, 162.09, 164.91, 167.12, 169.89 ppm; HRMS **(Fig. 23)**: Mass calculated for [$C_{19}H_{12}N_4O_7S$]: 440.39 (M^+) ; Obs. Mass: 440.38 (M^+)

Entry 4f, Table 2: ethyl (*E*)-2-(2-(3-methyl-5-(6-nitro-2-oxo-2*H*- chromen-3-yl)-1*H*-pyrazol-1-yl)-4-oxothiazol-5(4*H*)-ylidene)acetate

Orange solid; Obs. m.p. >300 °C; IR **(Fig. 24)**: 3158, 3124, 2265, 1730, 1701, 1658, 1519, 1486, 1368, 1268, 1181, 1130, 1065, 923, 802, 737, 605 cm^{-1}; ^1H NMR (400 MHz, DMSO-d_6) **(Fig. 25)**: δ 1.26-1.30 (t, 3H , -CH_2CH_3), 2.28 (s, 3H, -CH_3), 4.24- 4.29 (q, 2H, -CH_2), 6.68 (s, 1H, pyrazole - CH), 6.69 (s, 1H, vinyl -CH), 7.62-7.64 (d, Ar-1H, *J* = 9.2 Hz), 8.21-8.24 (dd, Ar-1H, *J* = 3.2 Hz, 9.2 Hz), 8.75 (s, 1H, coumarin - CH), 8.98-8.99 (d, Ar-1H, *J* = 3.2 Hz); ^{13}C NMR (100 MHz, DMSO-d_6): (δ ppm) : Due to insufficient solubility we are unable to scan its ^{13}C NMR; HRMS **(Fig. 26)**: Mass calculated for [$C_{20}H_{14}N_4O_7S$]: 454.41 (M^+) ; Obs. Mass: 454.40 (M^+).

Entry 4g, Table 2 : methyl (*E*)-2-(2-(5-(6-bromo-2-oxo-2*H*-chromen-3-yl)-3-methyl-1*H*-pyrazol-1-yl)-4-oxothiazol-5(4*H*)- ylidene)acetate

Orange solid; Obs. m.p. 300 °C; IR **(Fig. 27)**: 3270, 3060, 1923, 1836, 1780, 1731, 1698, 1595, 1551, 1512, 1470, 1403, 1328,1285, 1240, 1189, 1114, 1067, 1013, 930, 877, 803, 673, 593 cm^{-1}; ^1H NMR (400 MHz, DMSO-d_6) **(Fig. 28)**: δ 2.26 (s, 3H, -CH_3), 3.80 (s, 3H, -OCH_3), 6.69 (s, 1H, pyrazole -CH), 6.74 (s, 1H, vinyl -CH), 7.40-7.42 (d, Ar-1H, *J* = 9.2 Hz), 7.80-7.83 (dd, Ar-1H, *J* = 2.4 Hz, 8.8 Hz), 8.23-8.24 (d, Ar-1H, *J* = 2 Hz), 8.74 (s, 1H, coumarin -CH) ppm; ^{13}C NMR (100 MHz, DMSO-d_6): (δ ppm): Due to insufficient solubility we are unable to scan its ^{13}C NMR; HRMS **(Fig. 29)**: Mass calculated for [$C_{19}H_{12}BrN_3O_5S$]: 474.29 (M^+) ; Obs. Mass: 474.28 (M^+).

Entry 4h, Table 2 : methyl (*E*)-2-(2-(5-(6-chloro-2-oxo-2*H*-chromen-3-yl)-3methyl-1*H*-pyrazol-1-yl)-4-oxothiazol-5(4*H*)-ylidene)acetate

Orange solid; Obs. m.p. 286 °C; IR **(Fig. 30)**: 3066, 1781, 1734, 1696, 1596, 1551, 1524, 1469, 1415, 1328, 1285, 1239, 1189, 1119, 1073, 1013, 931, 875, 803, 774, 701, 677, 616 cm^{-1}; ^1H NMR (400 MHz, DMSO-d_6) **(Fig. 31)**: δ 2.23 (s, 3H, -CH_3), 3.76 (s, 3H, -OCH_3), 6.64 (s, 1H, pyrazol -CH), 6.70 (s, 1H, vinyl -CH), 7.40-7.42 (d, Ar-1H, *J* = 9.2 Hz), 7.56-7.58 (dd, Ar-1H, *J* =2.4 Hz, 8.8 Hz), 8.06-8.07 (d, Ar-1H, *J* =1.6 Hz), 8.70 (s, 1H, coumarin -CH) ppm; ^{13}C NMR (100 MHz, DMSO-d_6): (δ ppm): Due to insufficient

solubility we are unable to scan its ^{13}C NMR; HRMS **(Fig. 32)**: Mass calculated for [$C_{19}H_{12}ClN_3O_5S$]: 429.83 (M^+); Obs. Mass: 429.82 (M^+).

Entry 4i, Table 2 : methyl (*E*)-2-(2-(5-(6-bromo-8-methoxy-2-oxo-2*H*-chromen-3-yl)-3-methyl-1*H*-pyrazol-1-yl)-4-oxothiazol-5(4*H*)- ylidene)acetate

Orange solid; Obs. m.p. 278 °C; IR **(Fig. 33)**: 2973, 2784, 1719, 1636, 1497, 1444, 1325, 1252, 1096, 1036, 930, 813, 733, 616 cm^{-1}; ^1H NMR (400 MHz, DMSO-d_6) **(Fig. 34)**: δ 2.25 (s, 3H, -CH_3), 3.80 (s, 3H, -OCH_3), 3.94 (s, 3H, -OCH_3), 6.69 (s, 1H, pyrazol -CH), 6.73 (s, 1H, vinyl -CH), 7.48-7.48 (d, Ar-1H, *J* = 2Hz), 7.77-7.78 (d, Ar-1H, *J* = 1.6 Hz), 8.69 (s, 1H, coumarin -CH) ppm; ^{13}C NMR (100 MHz, DMSO-d_6) **(Fig. 35)**: δ 15.60, 56.86, 57.80, 105.96, 120.18, 121.53, 121.72, 123.39, 123.62, 125.82, 125.99, 139.93, 141.81, 151.30, 157.37, 159.83, 163.01, 169.19, 170.31 ppm; HRMS **(Fig. 36)**: Mass calculated for [$C_{20}H_{14}BrN_3O_6S$]: 504.31 (M^+) ; Obs. Mass: 504.30 (M^+).

Entry 4j, Table 2 : methyl (*E*)-2-(2-(3-methyl-5-(2-oxo-2*H*-benzo[g]chromen-3-yl)-1*H*-pyrazol-1-yl)-4-oxothiazol-5(4*H*)- ylidene)acetate

Orange solid; Obs. m.p. >300 °C; IR **(Fig. 37)**: 2965, 2772, 1766, 1709, 1641, 1589, 1491, 1443, 1408, 1309, 1247, 1072, 1039, 968, 833, 787, 755, 733, 694, 628 cm^{-1}; 1H NMR (400 MHz, DMSO-d_6) **(Fig. 38)**: δ 2.29 (s, 3H, -CH_3), 3.82 (s, 3H, -OCH_3), 6.73 (s, 1H, pyrazol -CH), 6.76 (s, 1H, vinyl -CH), 7.59-7.67 (m, Ar-2H), 7.75- 7.79 (m, Ar-1H), 8.06-8.08 (d, Ar-1H, *J* = 8 Hz), 8.10-8.29 (m, Ar-1H), 8.58-8.66 (m, Ar-1H), 9.30 (s, 1H, coumarin -CH) ppm; ^{13}C NMR (100 MHz, DMSO-d_6) **(Fig. 39)**: δ 15.89, 52.06, 100.42, 114.81, 115.80, 115.98, 116.15, 118.59, 118.77, 124.82, 126.10, 129.20, 129.79, 132.29, 140.71, 141.77, 142.72, 146.05, 153.06, 153.24, 161.09, 163.91, 166.12, 170.89 ppm; HRMS **(Fig. 40)**: Mass calculated for [$C_{23}H_{15}N_3O_5S$]: 445.45 (M^+); Obs. Mass: 445.44 (M^+).

IR, ^{1}H and ^{13}C NMR spectra of synthesized novel Bronsted acidic surfactant catalyst, 4-hexadecyl-4-methylmorpholin-4- ium hydrogen sulphate, $[HDMM]^{+}$ $[HSO4]^{-}$.

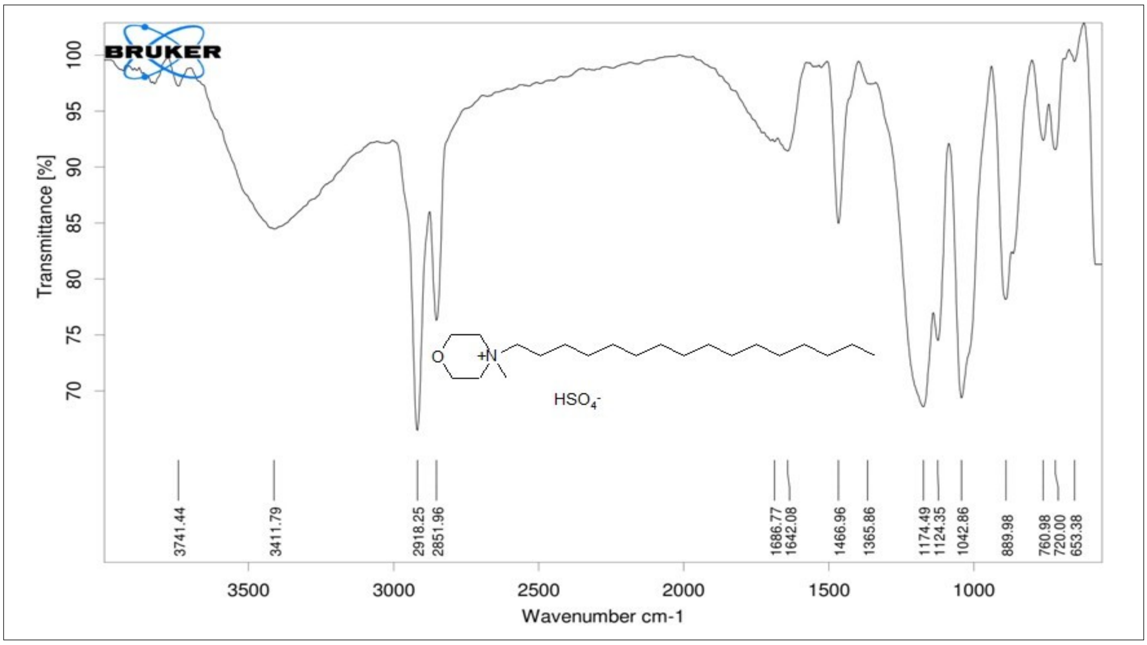

Fig. 1

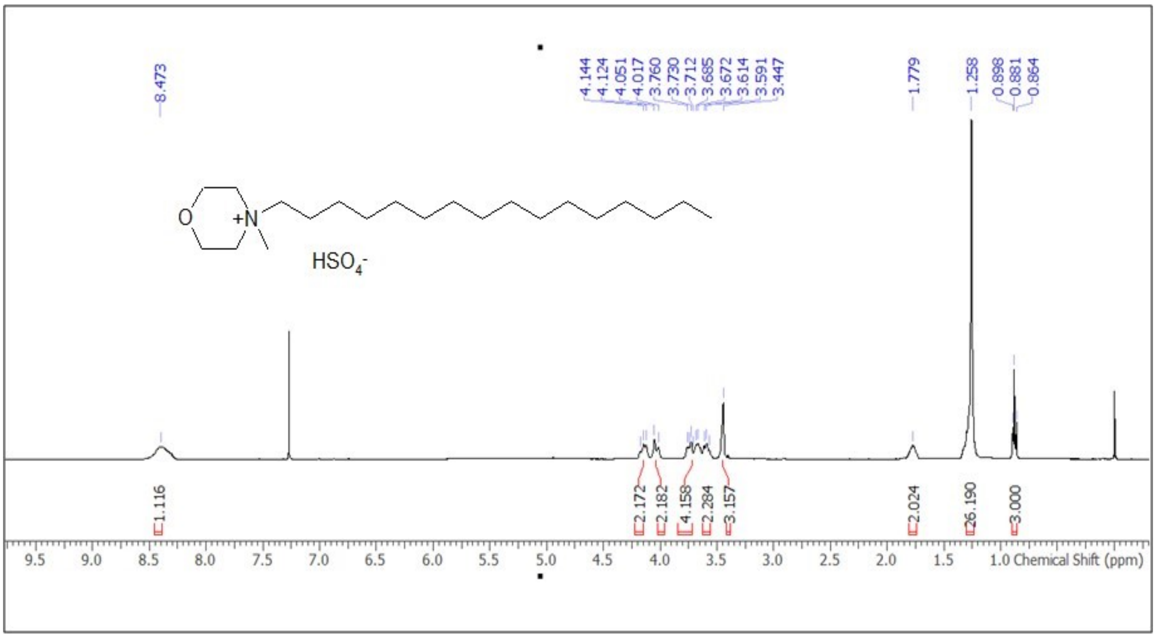

Fig. 2

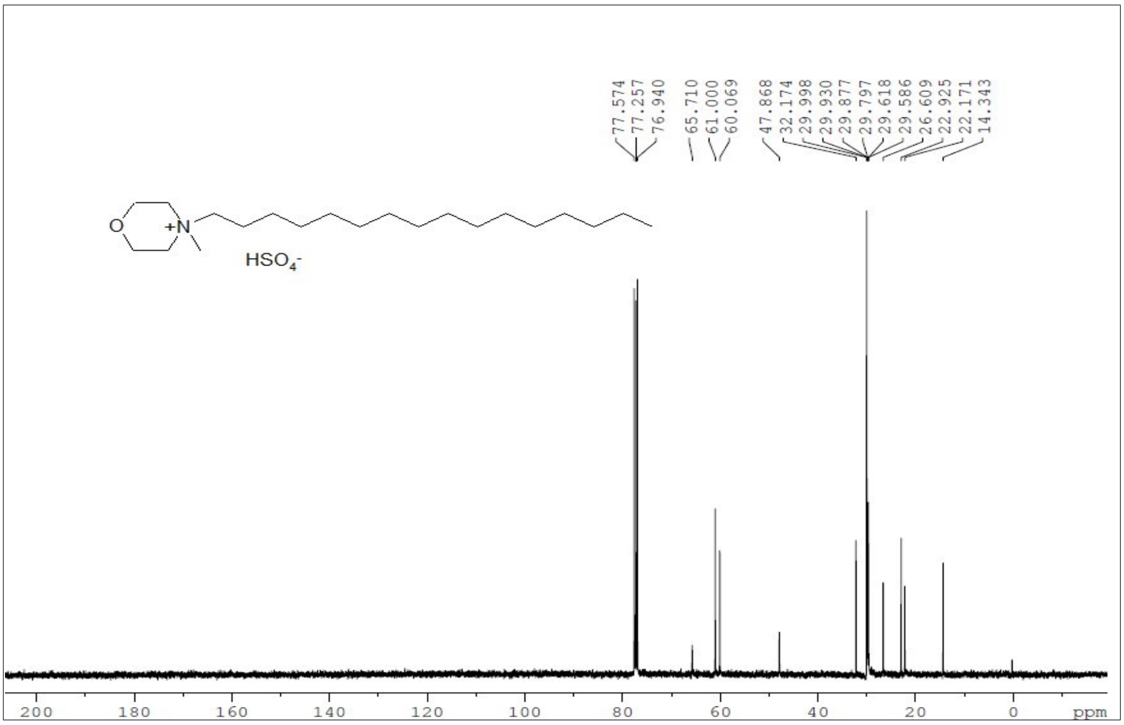
77.574
77.257
76.940
65.710
61.000
60.069
47.868
32.174
29.998
29.930
29.877
29.797
29.618
29.586
26.609
22.925
22.171
14.343
O
+N
HSO4-
200
180
160
140
120
100
80
60
40
20
0
ppm

Fig. 3

IR, ^{1}H, ^{13}C and Mass spectra of synthesized thiazolyl-pyrazole chromen-2-one derivatives

Entry 4a, Table 3:

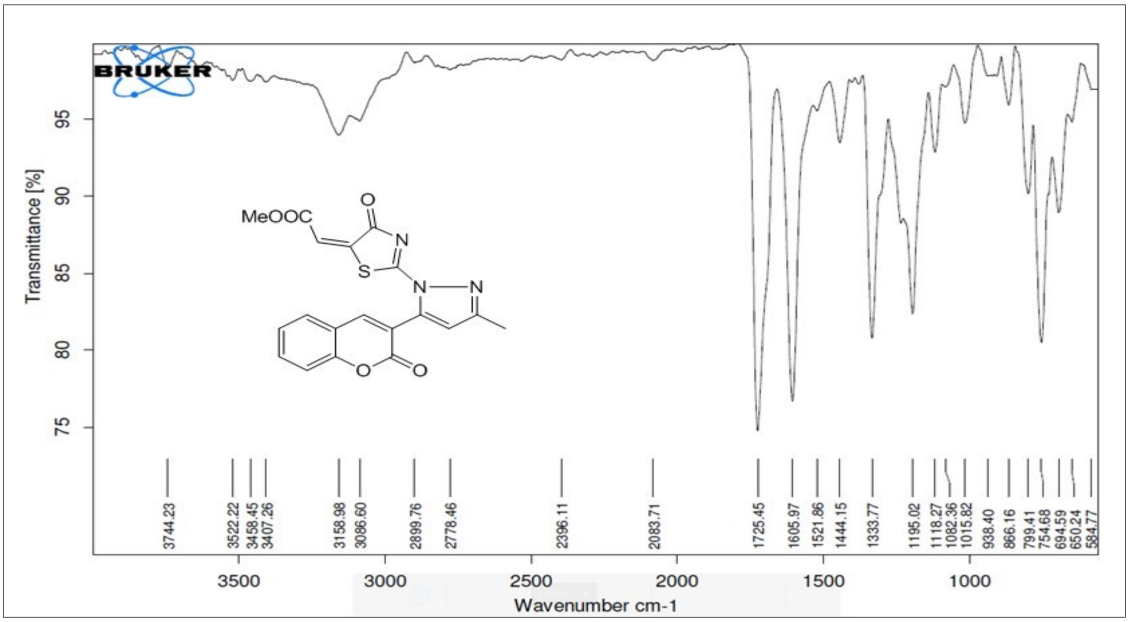

Fig. 4

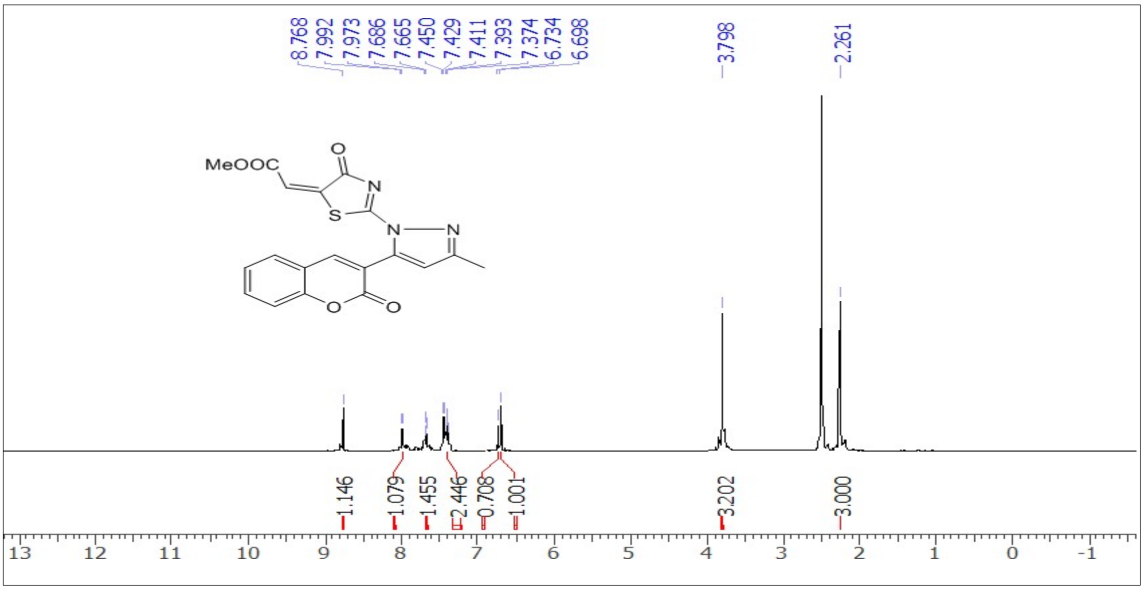

Fig. 5

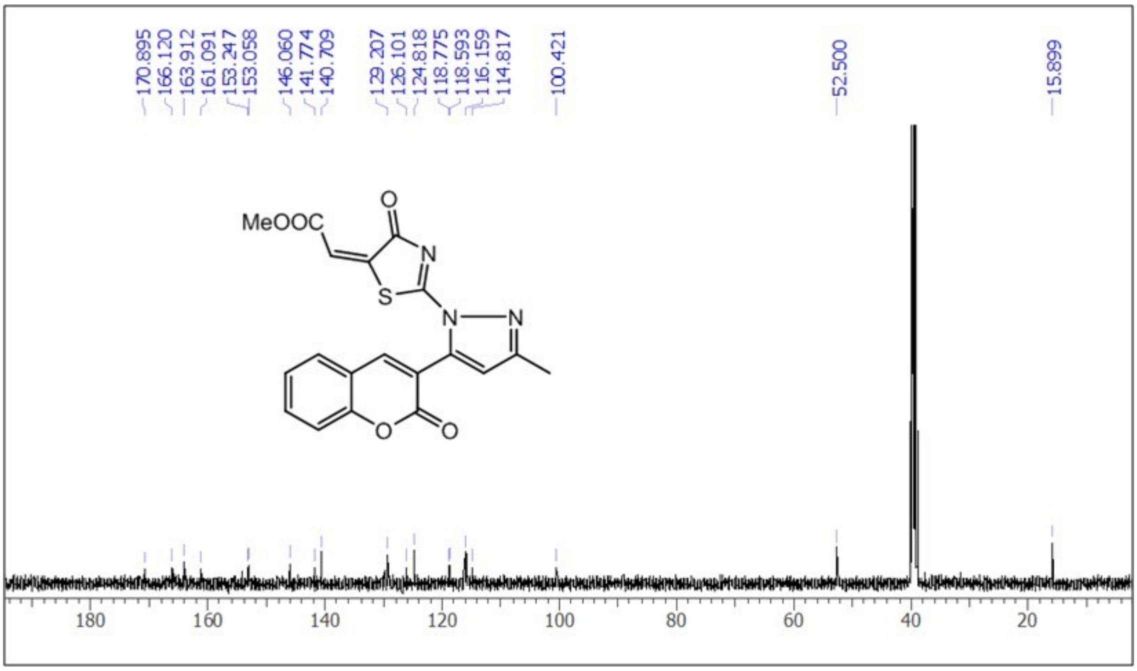
170.895
166.120
163.912
161.091
153.247
153.058
146.060
141.774
140.709
129.207
126.101
124.818
118.775
118.593
116.159
114.817
100.421
52.500
15.899
MeOOC
O
N
S
N
N
O
O
180
160
140
120
100
80
60
40
20

Fig. 6

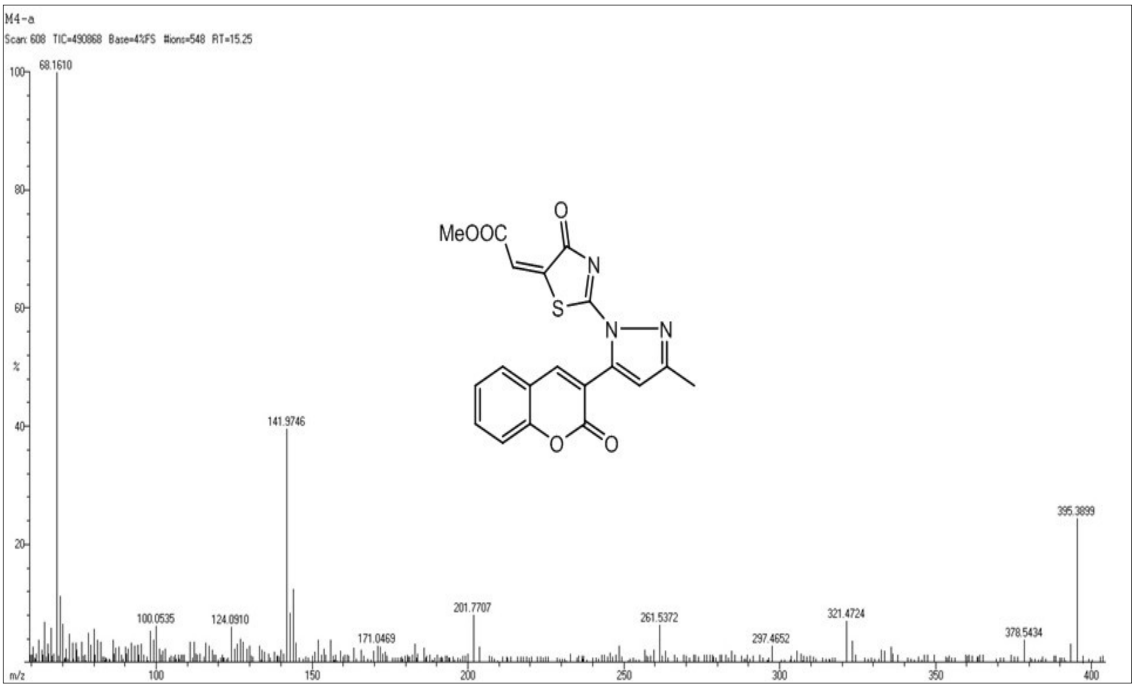
M4-a
Scan: 608 TIC=490868 Base=4%FS #ions=548 RT=15.25
68.1610
141.9746
395.3899
100.0535
124.0910
171.0469
201.7707
261.5372
297.4652
321.4724
378.5434
MeOOC
O
N
S
N
N
O
O
%
m/z

Fig. 7

Entry 4b, Table 3:

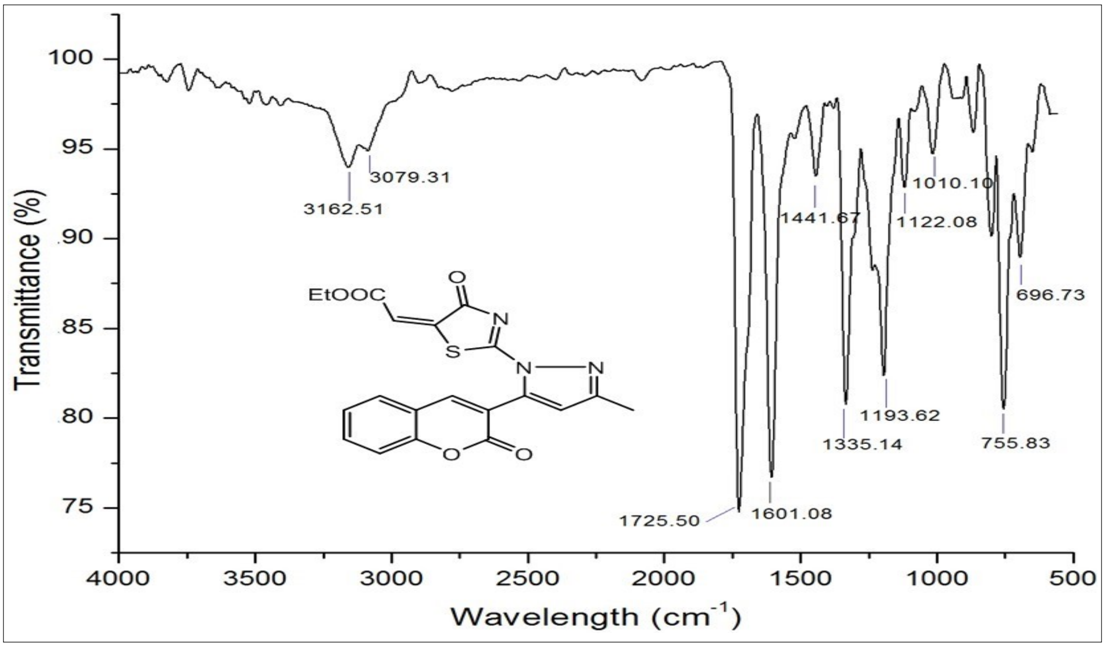

Fig. 8

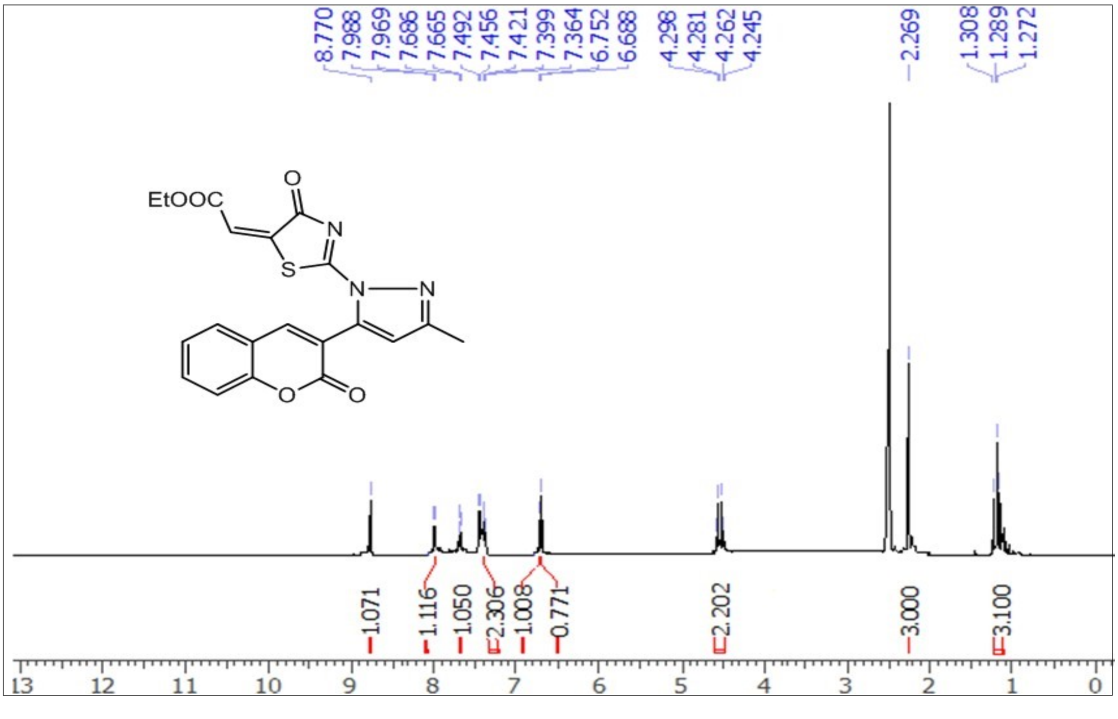

Fig. 9

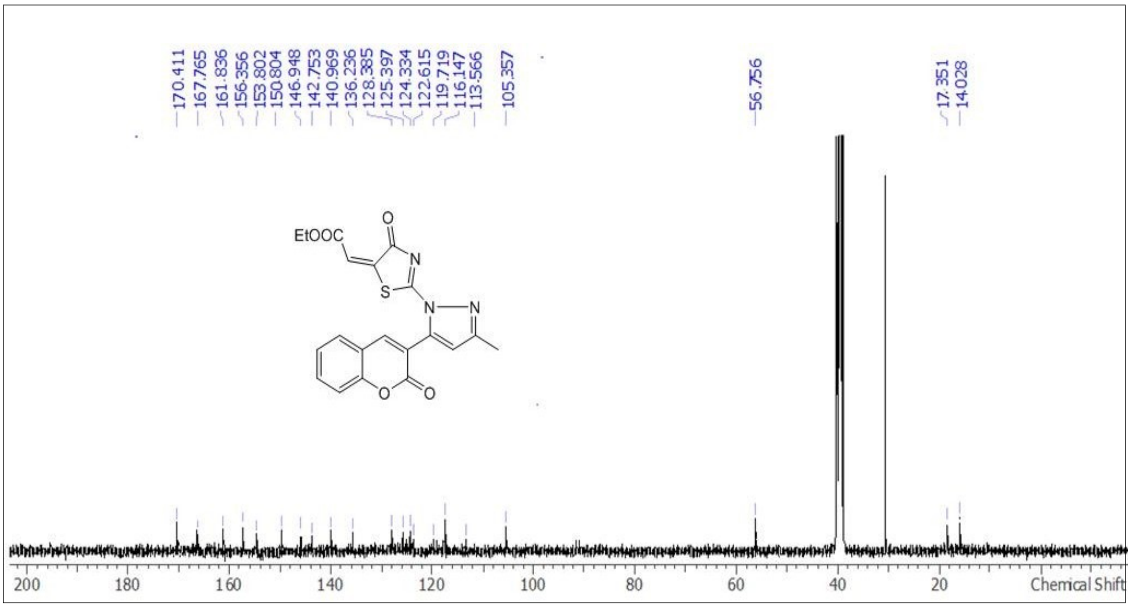
170.411
167.765
161.836
156.356
153.802
150.804
146.948
142.753
140.969
136.236
128.385
125.397
124.334
122.615
119.719
116.147
113.566
105.357
56.756
17.351
14.028
EtOOC
O
N
S
N
N
O
O
200
180
160
140
120
100
80
60
40
20
Chemical Shift

Fig. 10

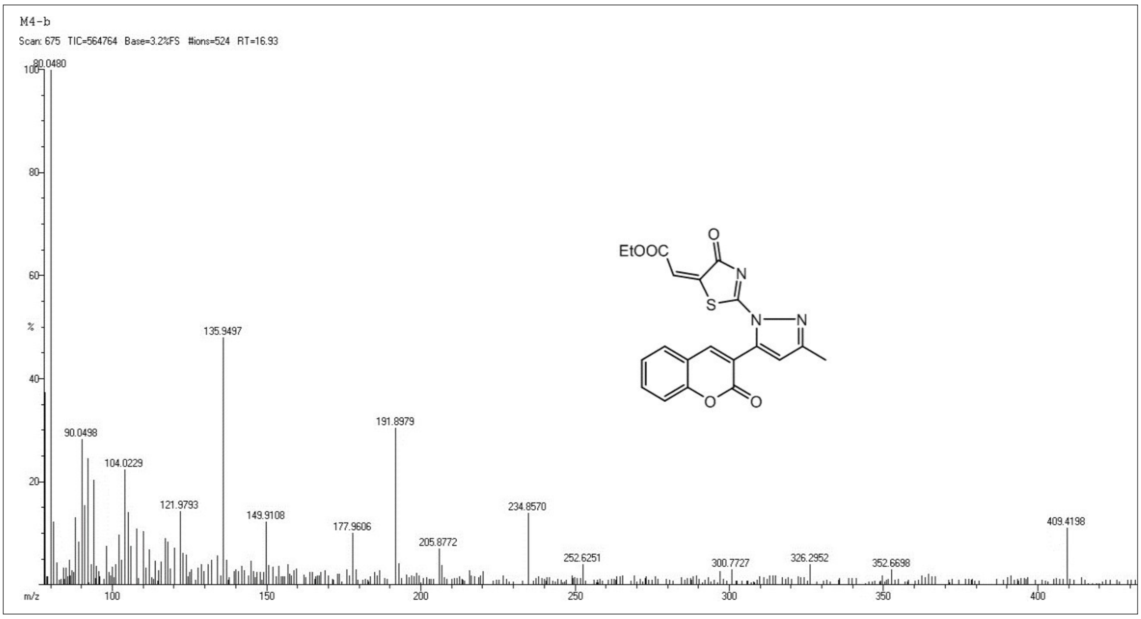
M4-b
Scan: 675 TIC=564764 Base=3.2%FS #ions=524 RT=16.93
80.0480
100
80
60
%
40
20
90.0498
104.0229
121.9793
135.9497
149.9108
177.9606
191.8979
205.8772
234.8570
252.6251
300.7727
326.2952
352.6698
409.4198
m/z
100
150
200
250
300
350
400
EtOOC
O
N
S
N
N
O
O

Fig. 11

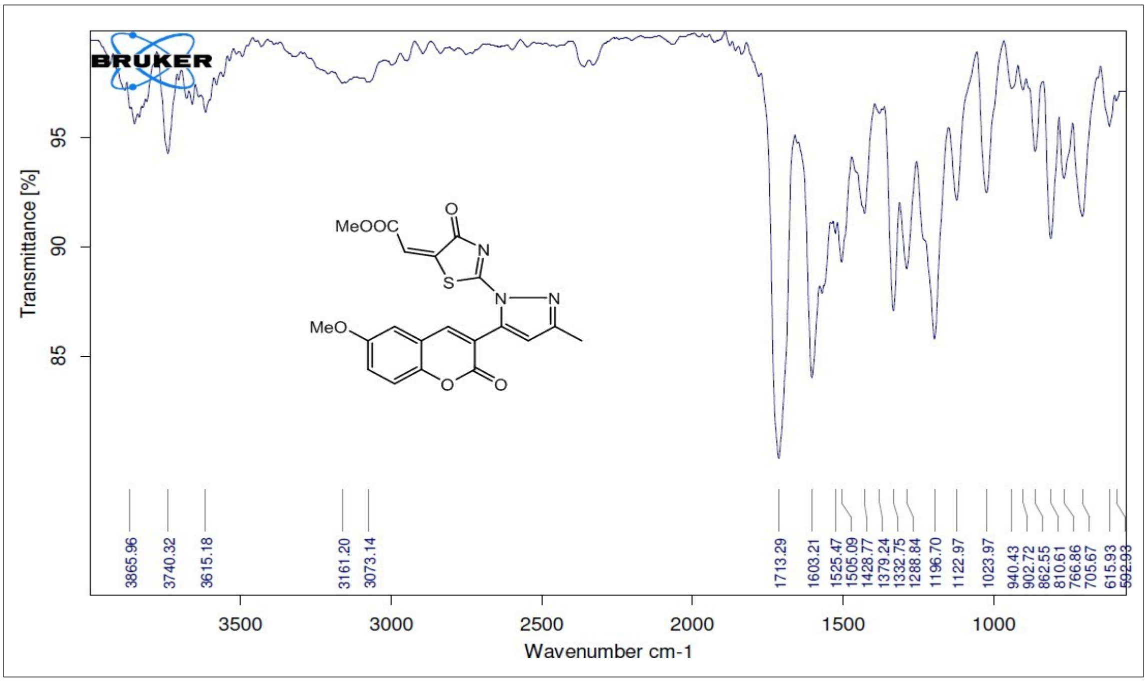
BRUKER
Transmittance [%]
95
90
85
MeOOC
O
N
S
N
N
MeO
O
O
3865.96
3740.32
3615.18
3161.20
3073.14
1713.29
1603.21
1525.47
1505.09
1428.77
1379.24
1332.75
1288.84
1196.70
1122.97
1023.97
940.43
902.72
862.55
810.61
766.86
705.67
615.93
592.93
3500
3000
2500
2000
1500
1000
Wavenumber cm-1

Fig. 12

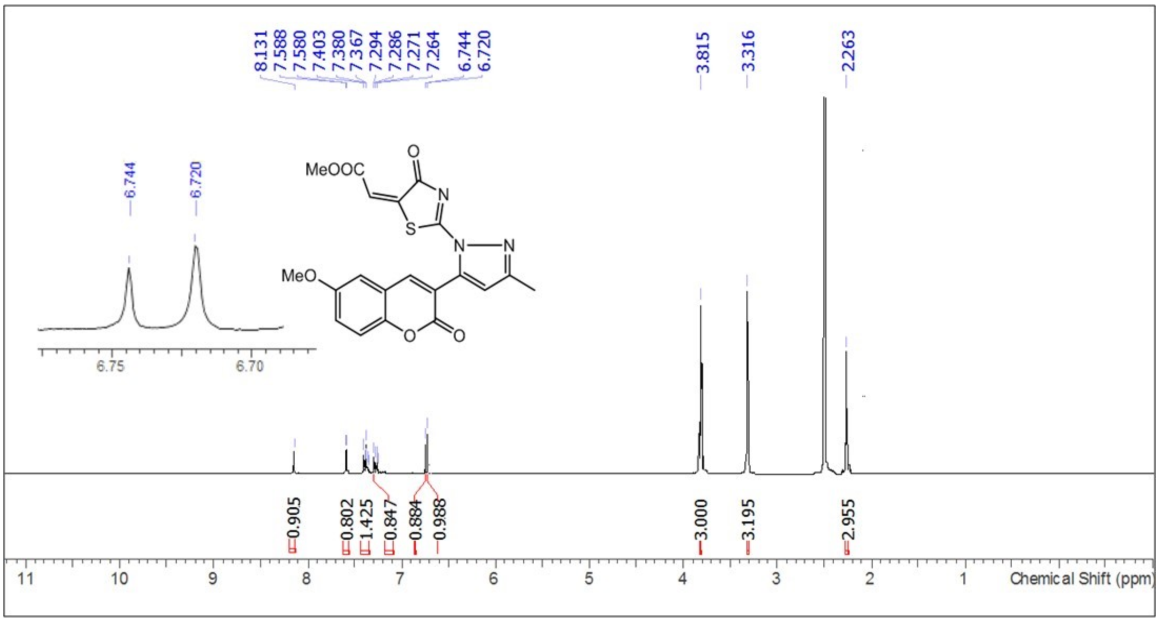
8.131
7.588
7.580
7.403
7.380
7.367
7.294
7.286
7.271
7.264
6.744
6.720
3.815
3.316
2.263
6.744
6.720
6.75
6.70
MeOOC
O
N
S
N
N
MeO
O
O
0.905
0.802
1.425
0.847
0.884
0.988
3.000
3.195
2.955
11
10
9
8
7
6
5
4
3
2
1
Chemical Shift (ppm)

Fig. 13

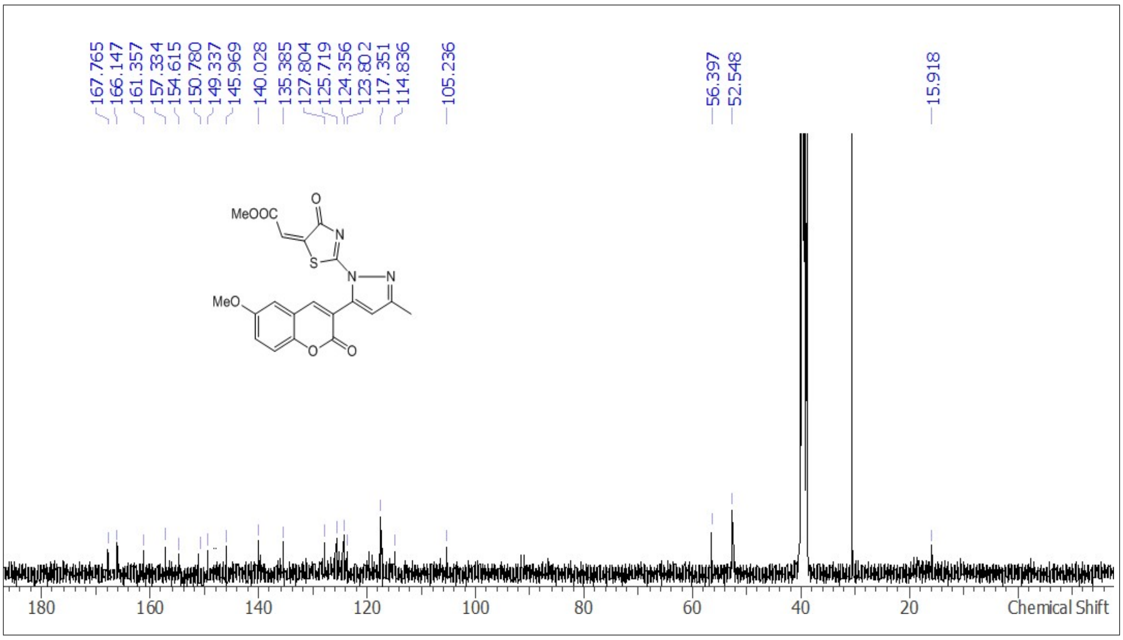
167.765
166.147
161.357
157.334
154.615
150.780
149.337
145.969
140.028
135.385
127.804
125.719
124.356
123.802
117.351
114.836
105.236
56.397
52.548
15.918
MeOOC
O
N
S
N
N
MeO
O
O
180
160
140
120
100
80
60
40
20
Chemical Shift

Fig. 14

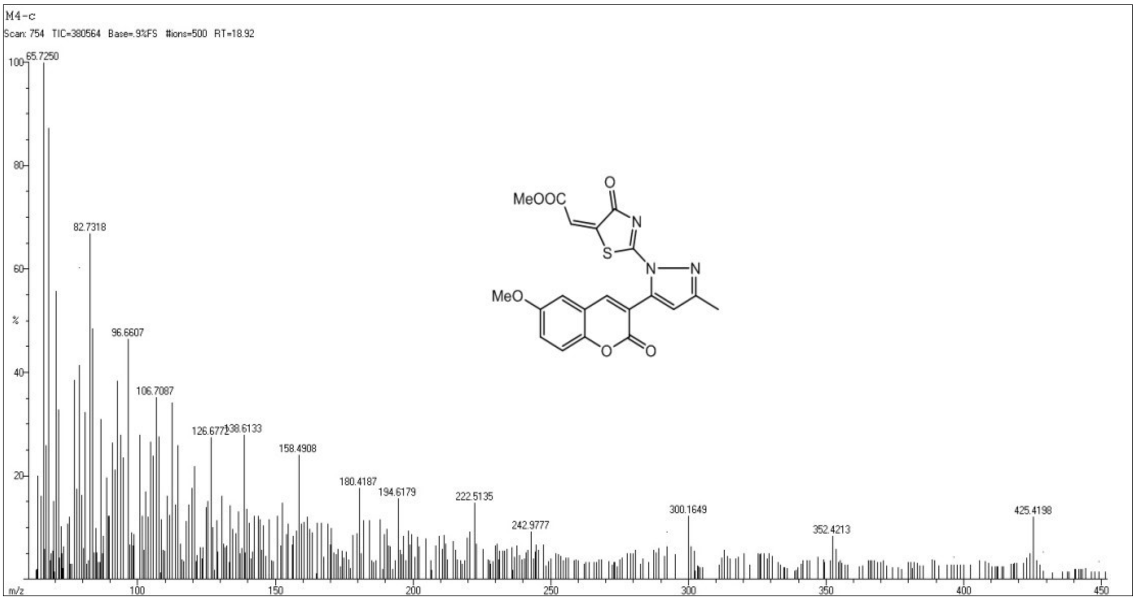
M4-c
Scan: 754 TIC=380564 Base=.9%FS #ions=500 RT=18.92
65.7250
82.7318
96.6607
106.7087
126.6772
138.6133
158.4908
180.4187
194.6179
222.5135
242.9777
300.1649
352.4213
425.4198
MeOOC
MeO
%
m/z

Fig. 15

Entry 4d, Table 3:

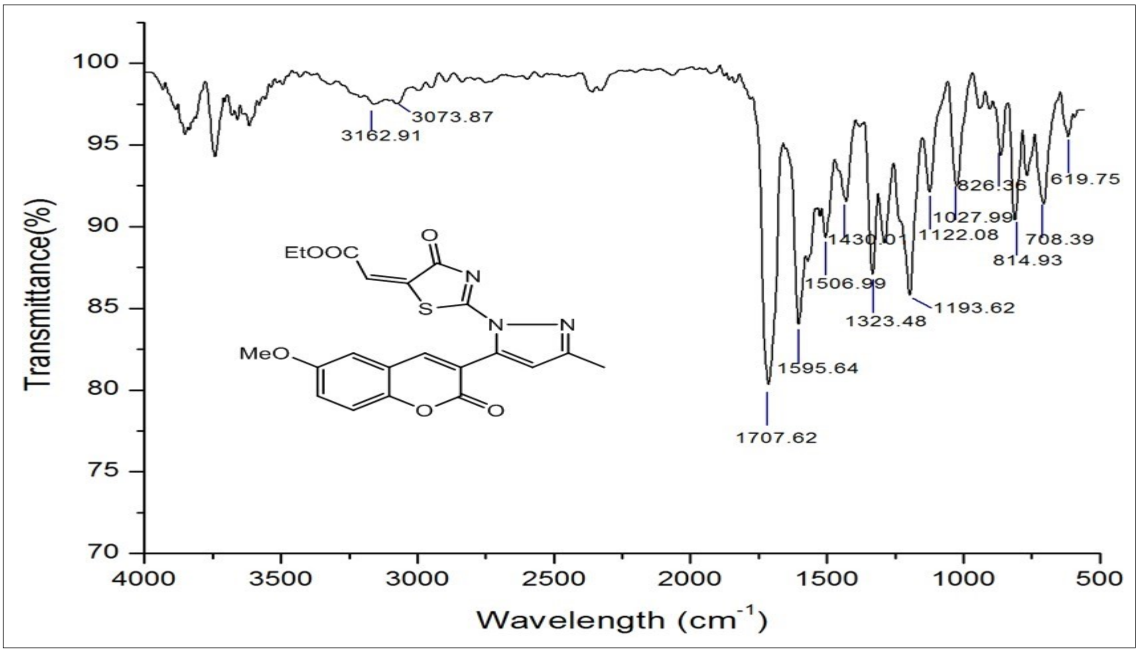

Fig. 16

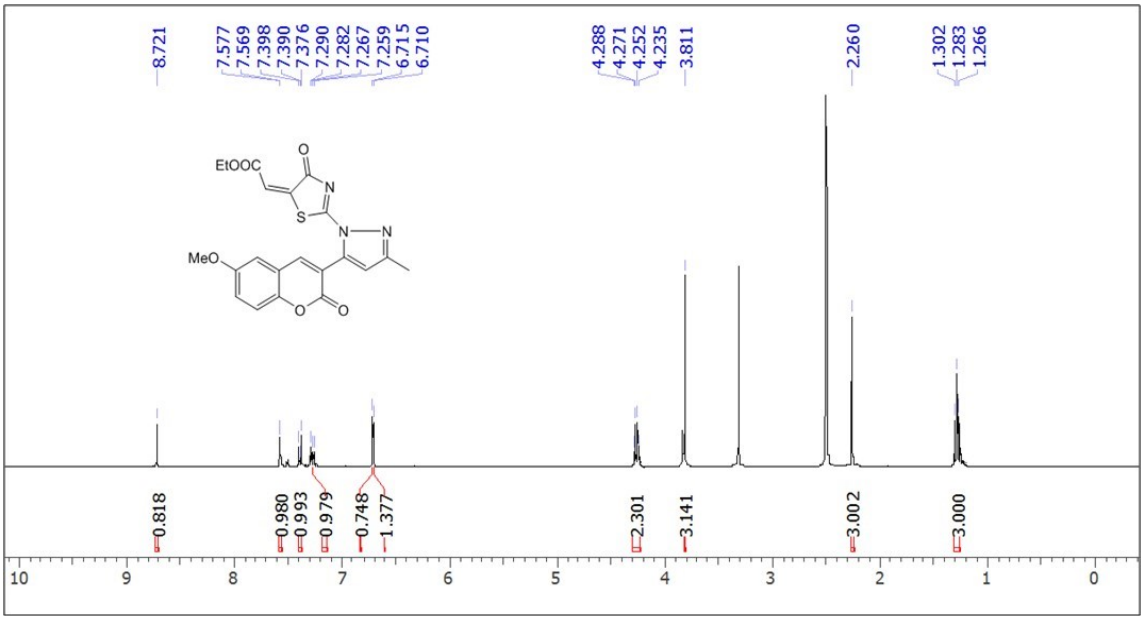

Fig. 17

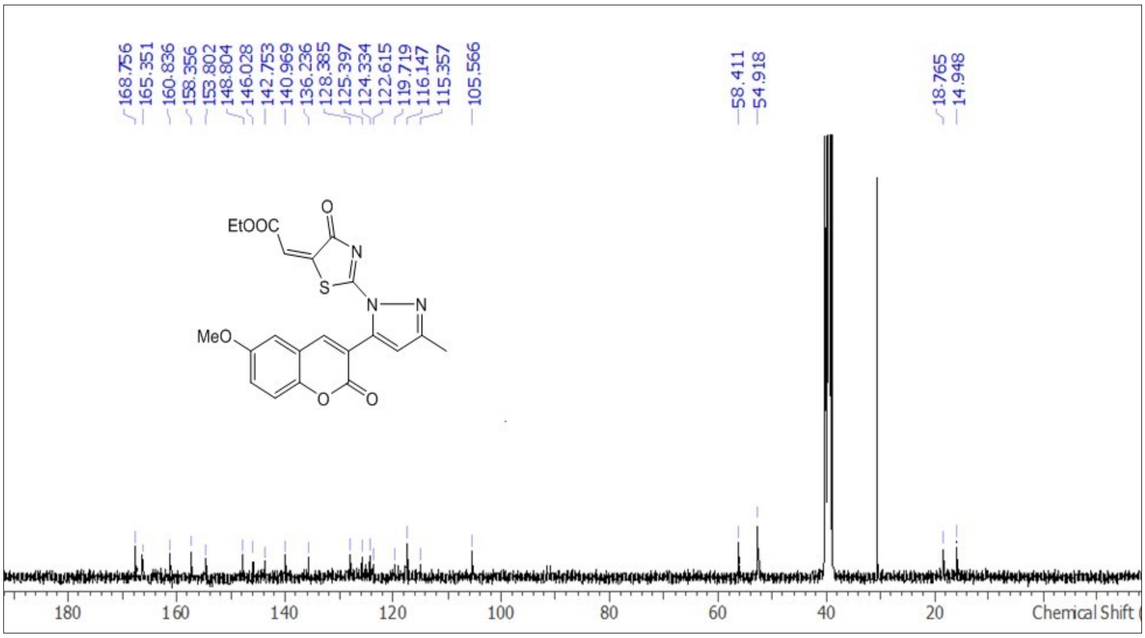

168.756
165.351
160.836
158.356
153.802
148.804
146.028
142.753
140.969
136.236
128.385
125.397
124.334
122.615
119.719
116.147
115.357
105.566
58.411
54.918
18.765
14.948
EtOOC
O
N
S
N
N
MeO
O
O
180
160
140
120
100
80
60
40
20
Chemical Shift (

Fig. 18

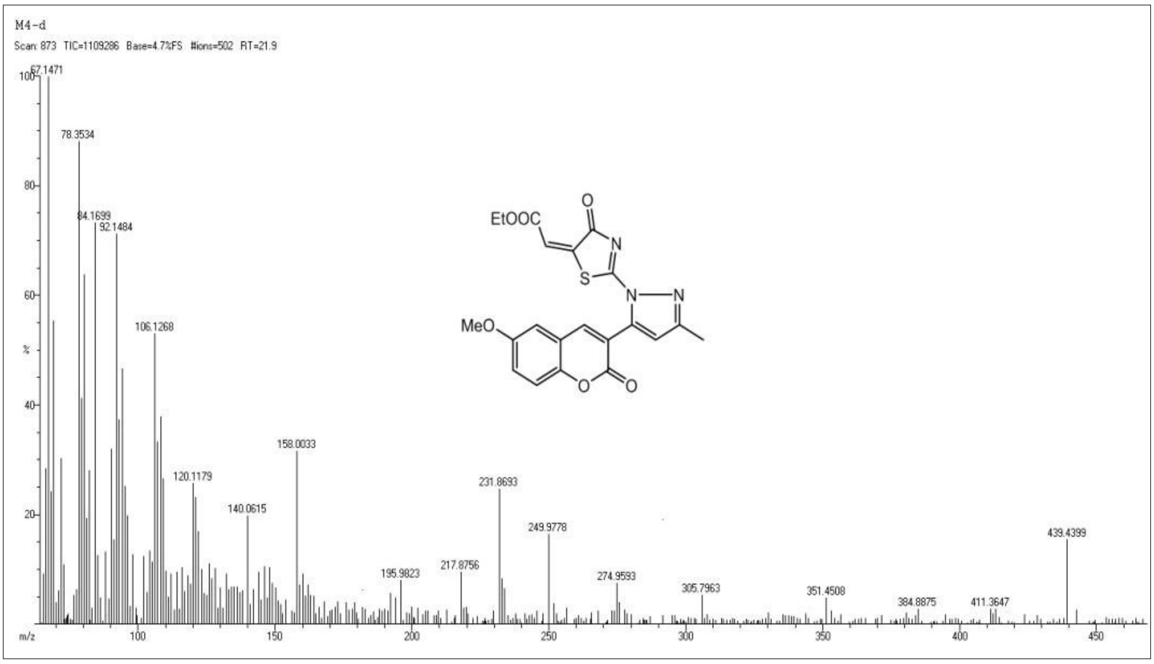

M4-d
Scan: 873 TIC=1109286 Base=4.7%FS #ions=502 RT=21.9
67.1471
78.3534
84.1699
92.1484
106.1268
120.1179
140.0615
158.0033
195.9823
217.8756
231.8693
249.9778
274.9593
305.7963
351.4508
384.8875
411.3647
439.4399
100
80
60
%
40
20
m/z
100
150
200
250
300
350
400
450
EtOOC
O
N
S
N
N
MeO
O
O

Fig. 19

Entry 4e, Table 3:

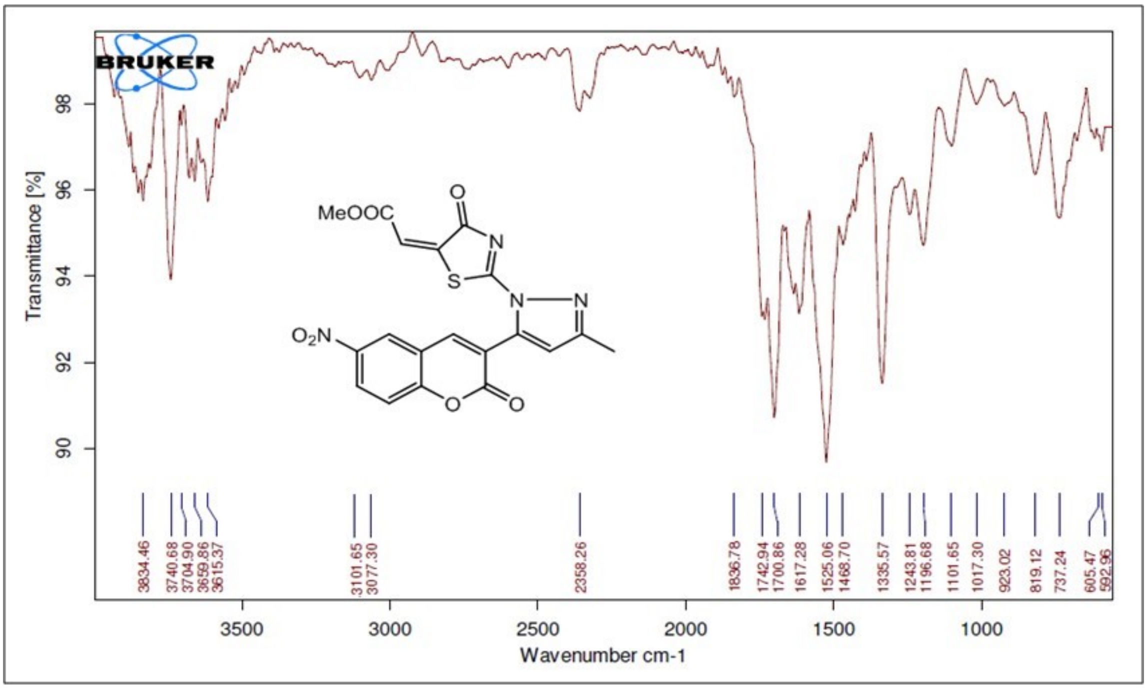

Fig. 20

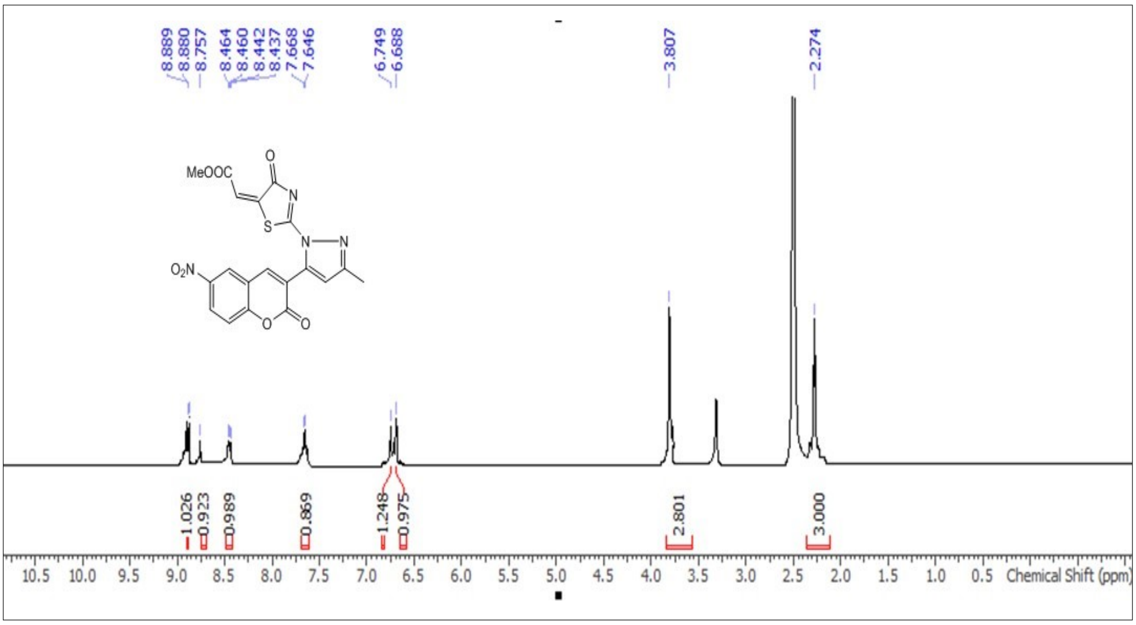

Fig. 21

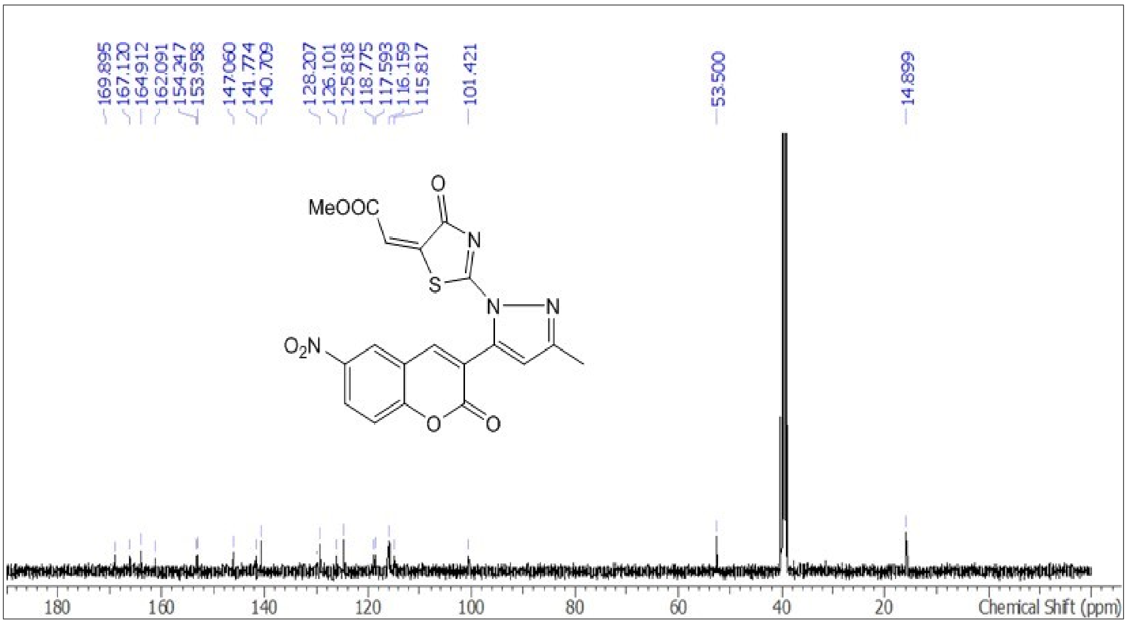

Fig. 22

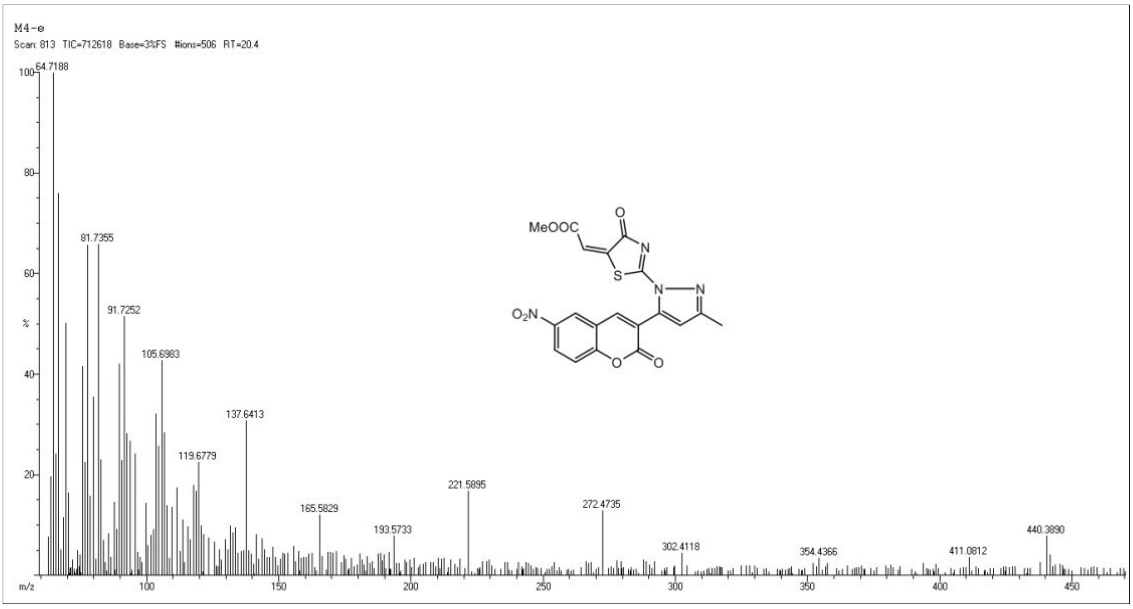

Fig. 23

Entry 4f, Table 3:

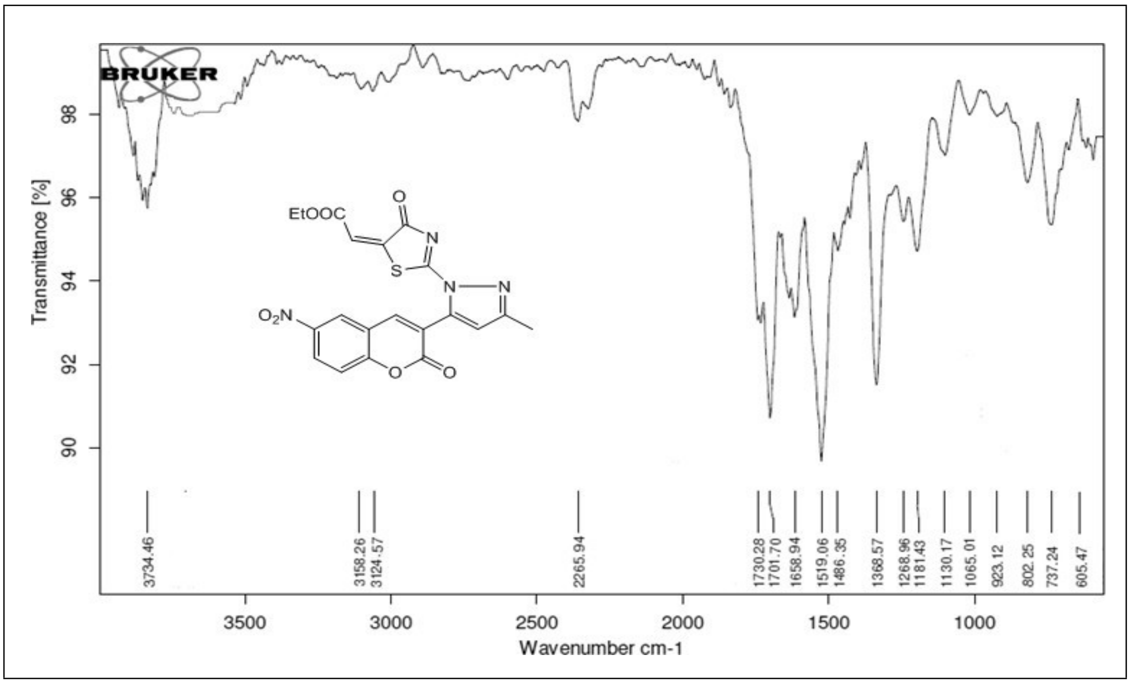

Fig. 24

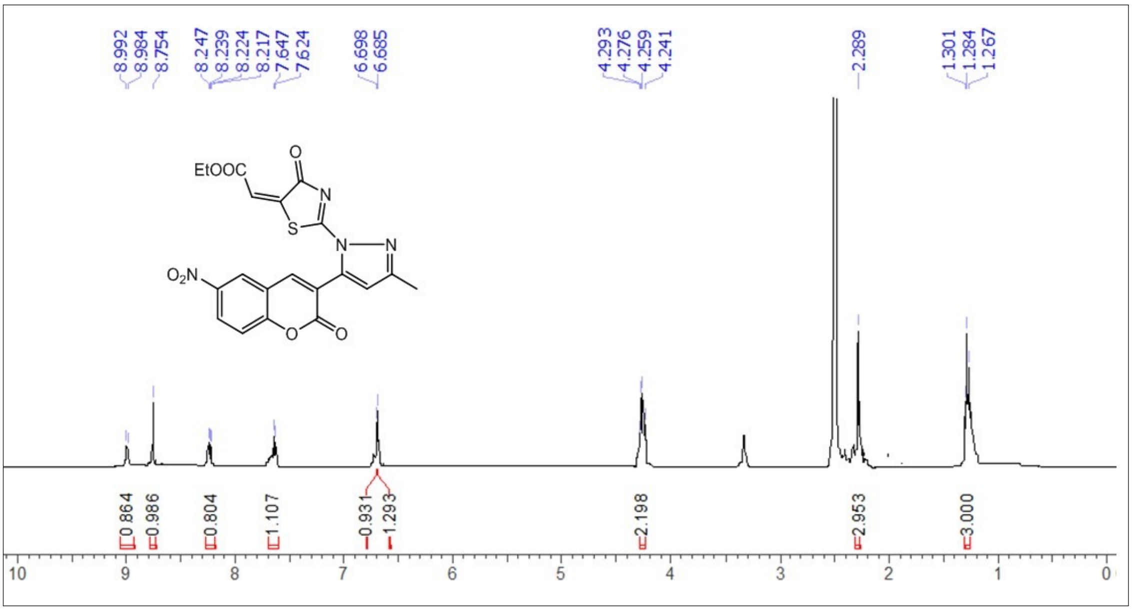

Fig. 25

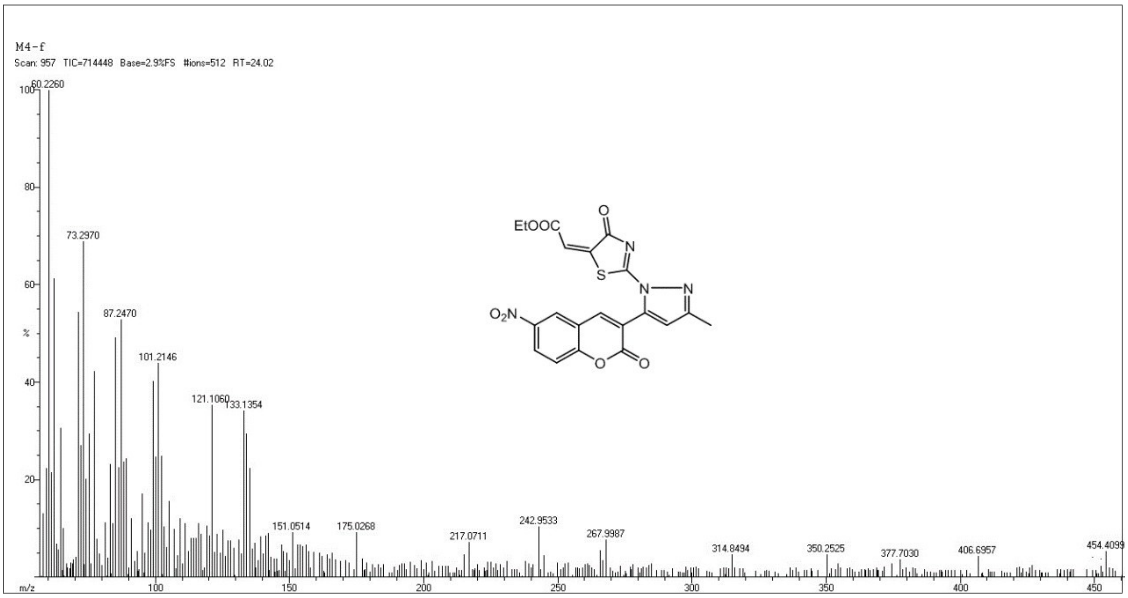

Fig. 26

Entry 4g, Table 3:

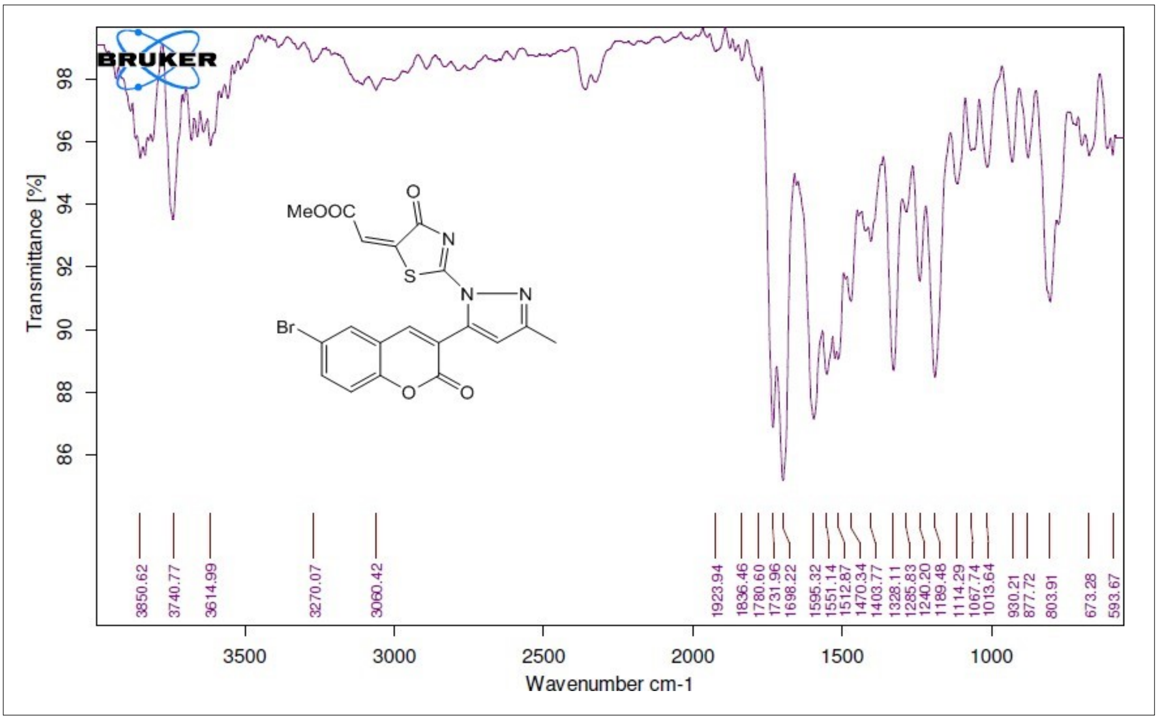

Fig. 27

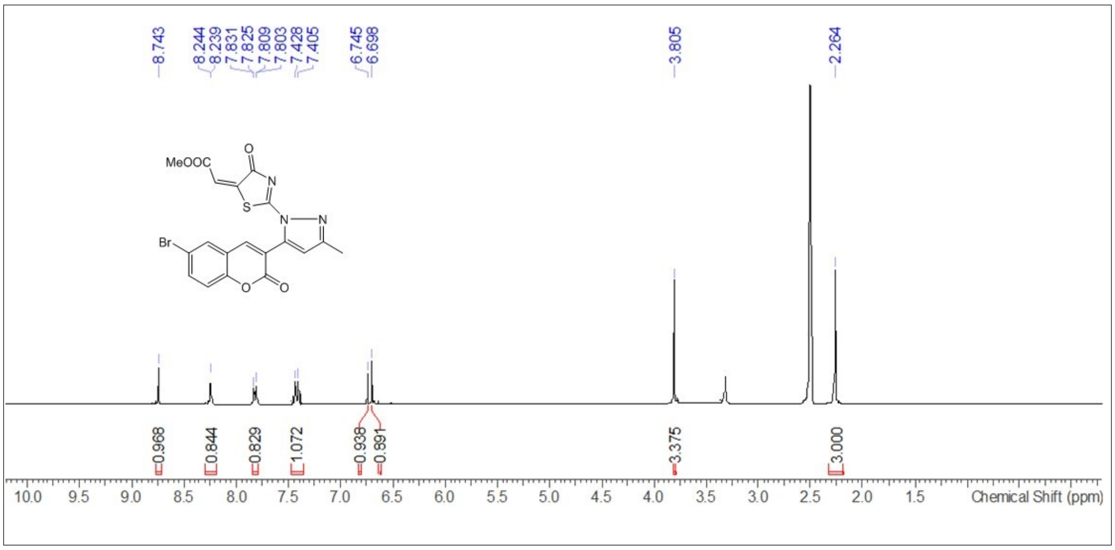
8.743
8.244
8.239
7.831
7.825
7.809
7.803
7.428
7.405
6.745
6.698
3.805
2.264
MeOOC
O
N
S
N
N
Br
O
O
0.968
0.844
0.829
1.072
0.938
0.891
3.375
3.000
10.0 9.5 9.0 8.5 8.0 7.5 7.0 6.5 6.0 5.5 5.0 4.5 4.0 3.5 3.0 2.5 2.0 1.5
Chemical Shift (ppm)

Fig. 28

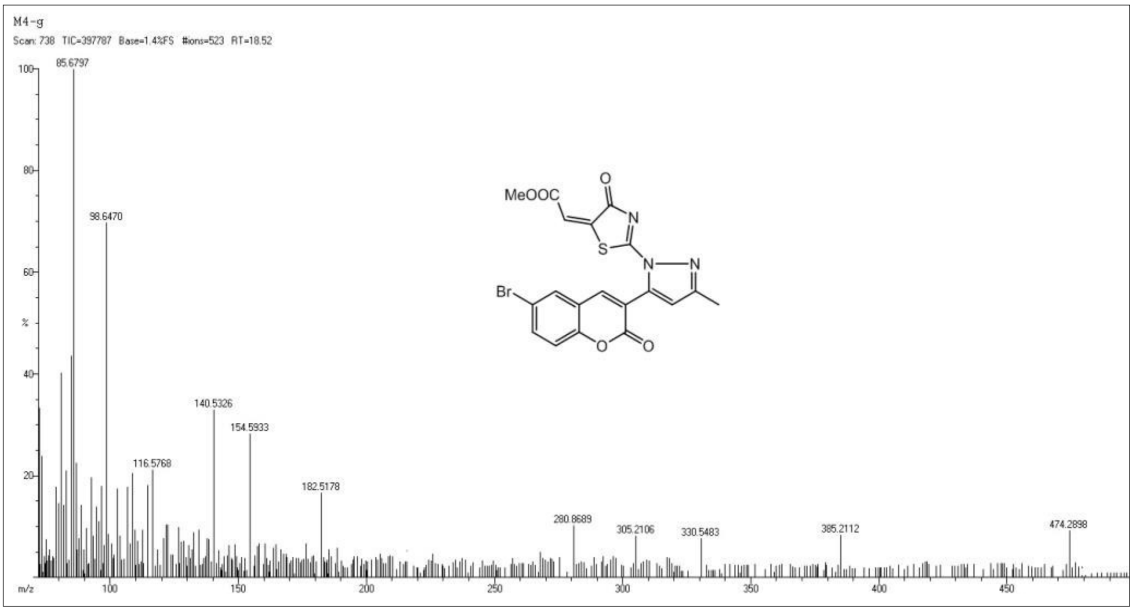
M4-g
Scan: 738 TIC=397787 Base=1.4%FS #ions=523 RT=18.52
85.6797
98.6470
140.5326
154.5933
116.5768
182.5178
280.8689
305.2106
330.5483
385.2112
474.2898
MeOOC
O
N
S
N
N
Br
O
O
%
m/z

Fig. 29

Entry 4h, Table 3:

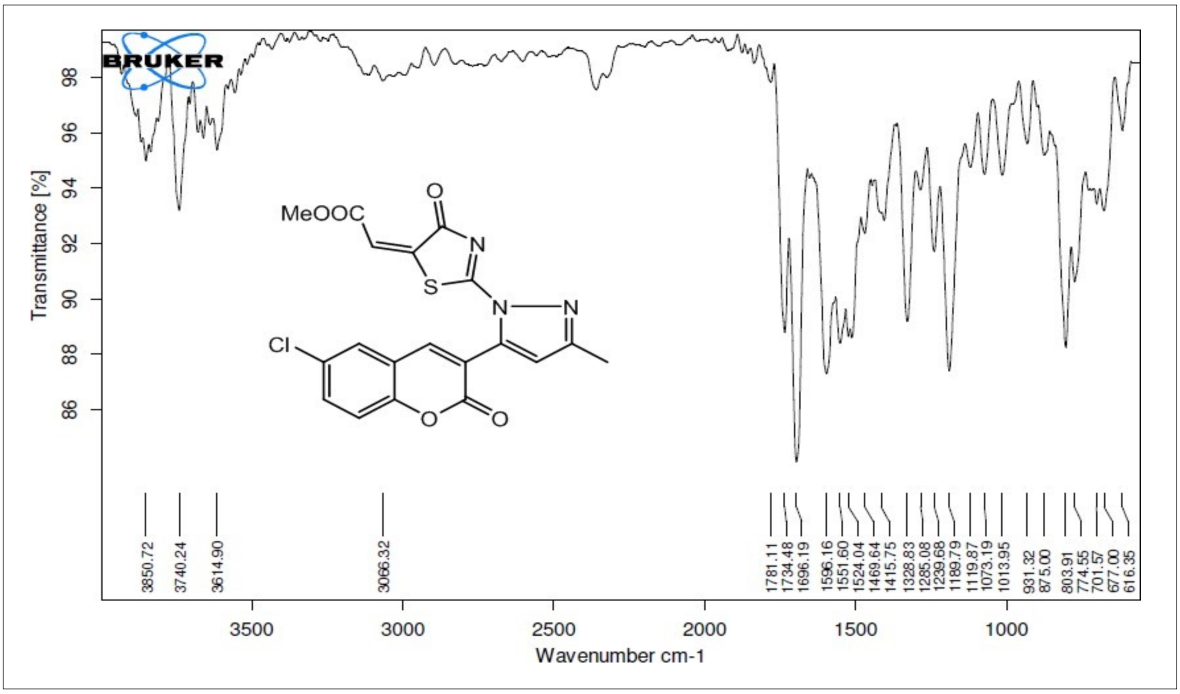

Fig. 30

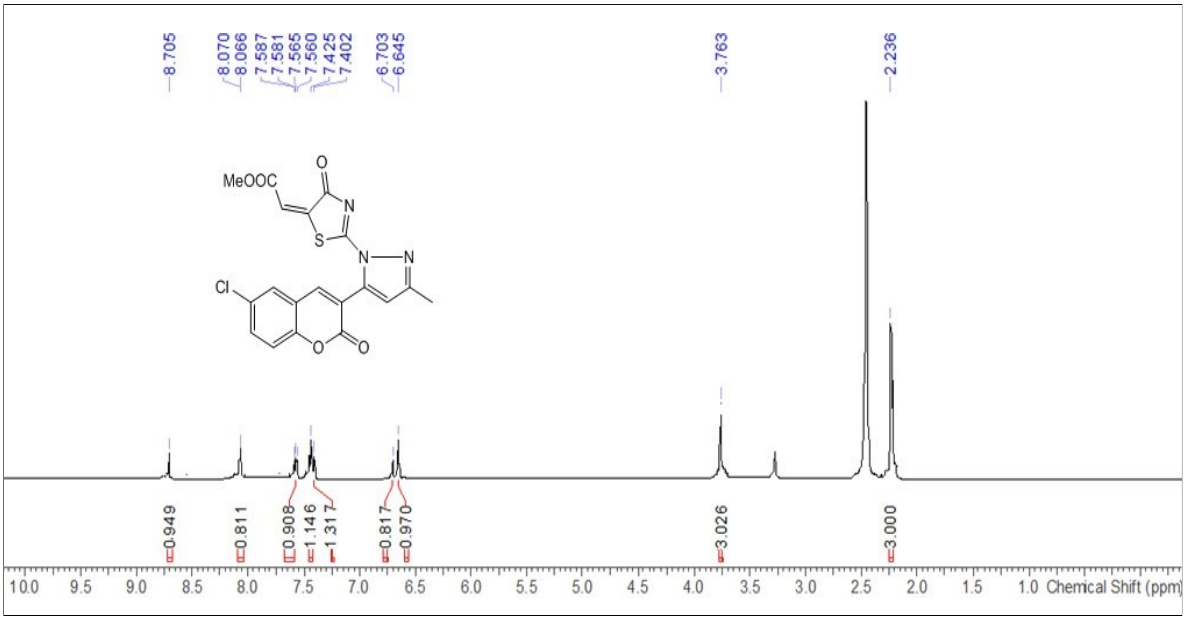

Fig. 31

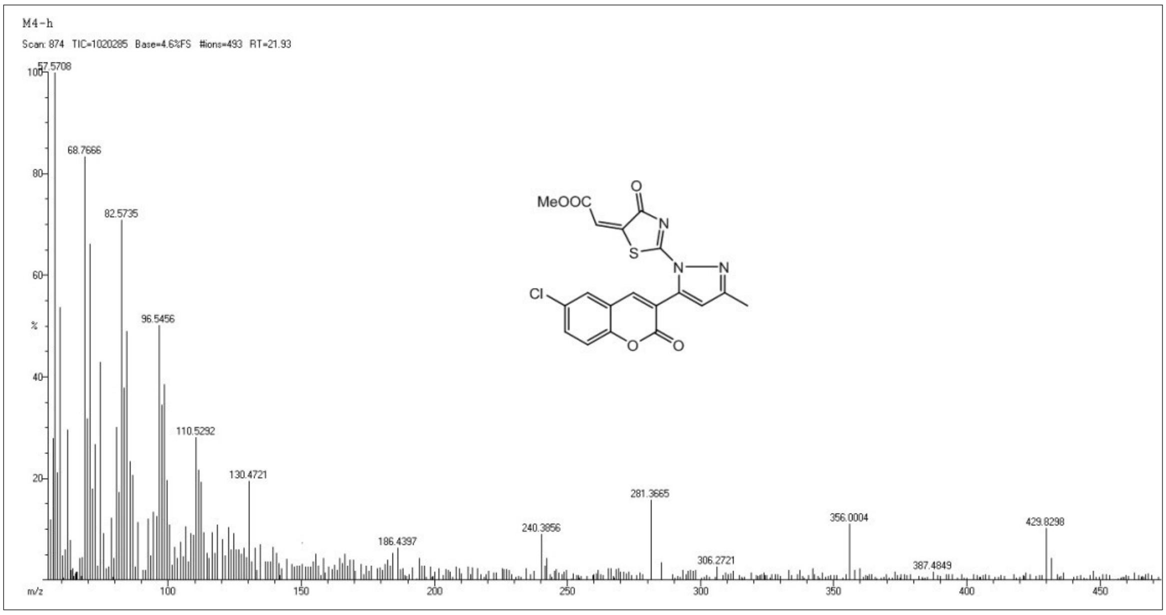

Fig. 32

Entry 4i, Table 3:

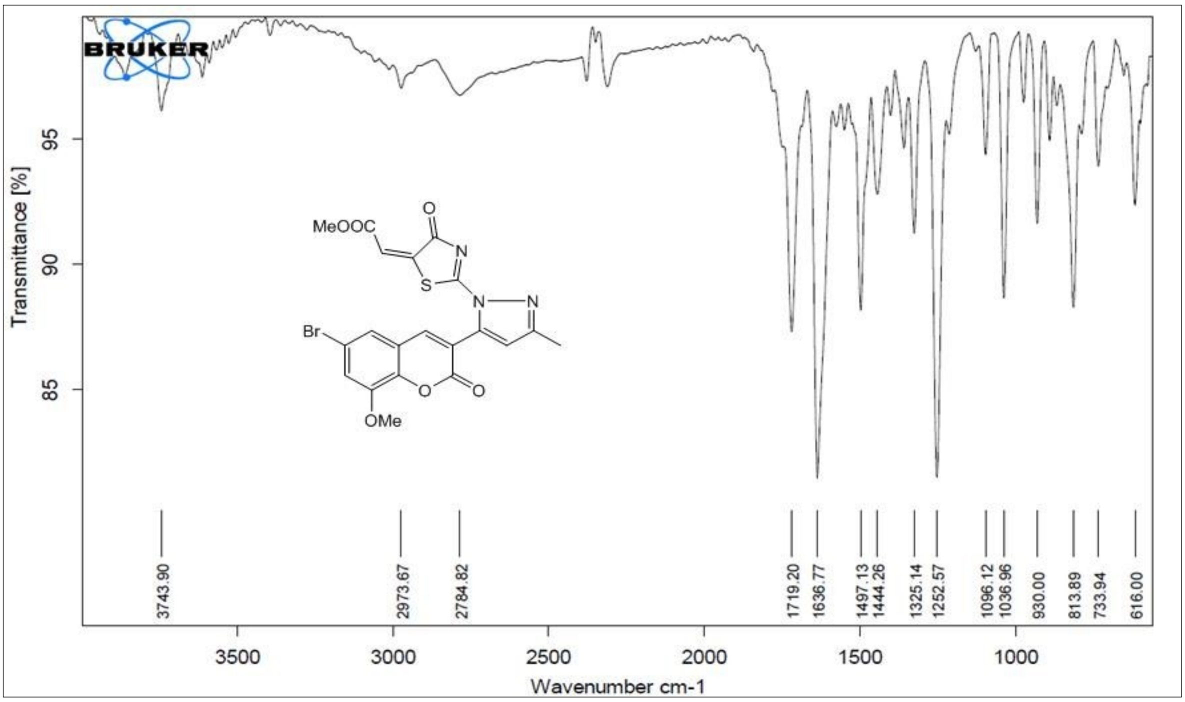

Fig. 33

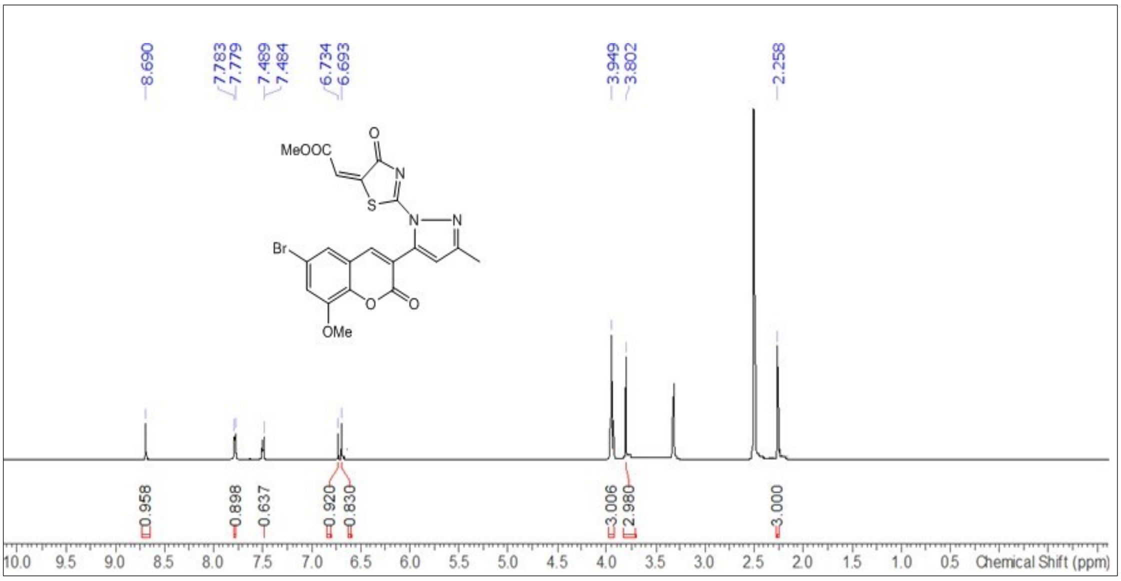
8.690
7.783
7.779
7.489
7.484
6.734
6.693
3.949
3.802
2.258
MeOOC
O
N
S
N
N
Br
O
O
OMe
0.958
0.898
0.637
0.920
0.830
3.006
2.980
3.000
10.0
9.5
9.0
8.5
8.0
7.5
7.0
6.5
6.0
5.5
5.0
4.5
4.0
3.5
3.0
2.5
2.0
1.5
1.0
0.5
Chemical Shift (ppm)

Fig. 34

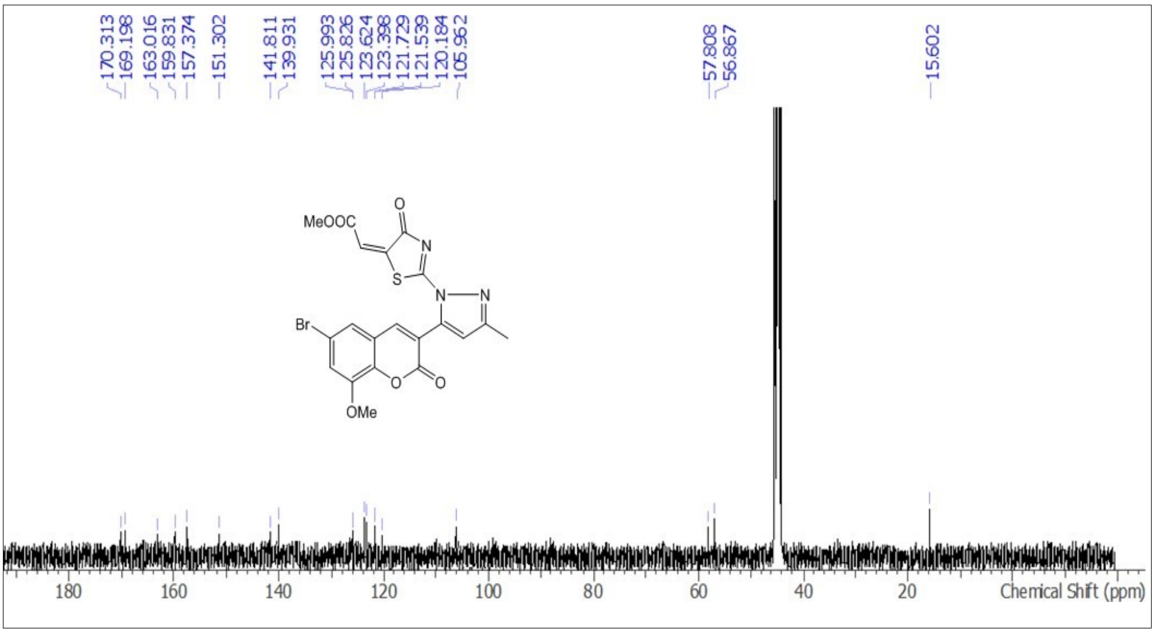
170.313
169.198
163.016
159.831
157.374
151.302
141.811
139.931
125.993
125.826
123.624
123.398
121.729
121.539
120.184
105.962
57.808
56.867
15.602
MeOOC
O
N
S
N
N
Br
O
O
OMe
180
160
140
120
100
80
60
40
20
Chemical Shift (ppm)

Fig. 35

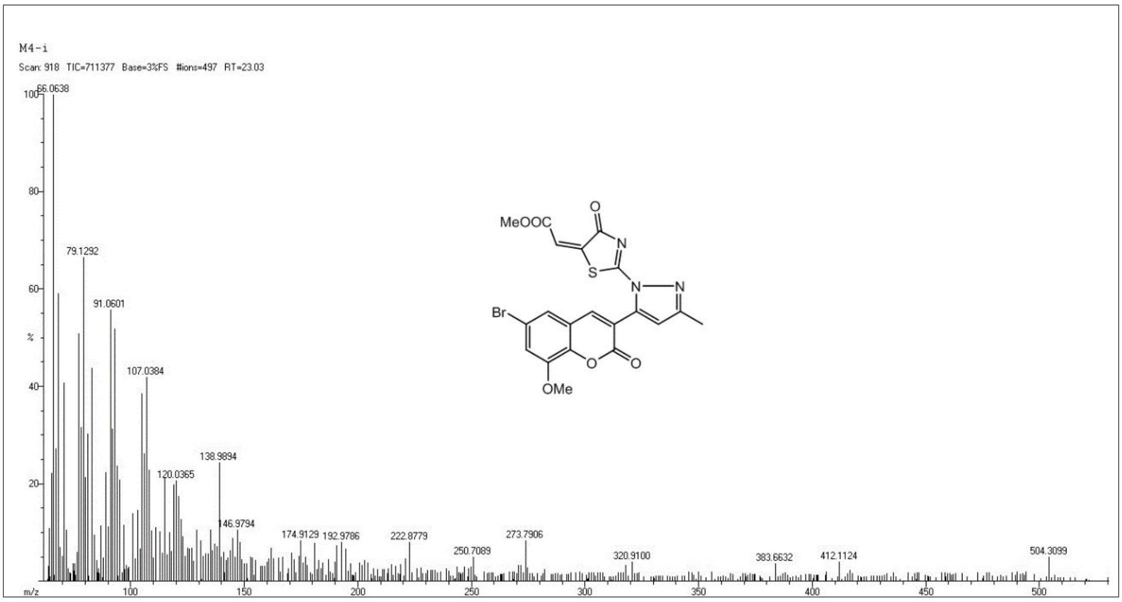

Fig. 36

Entry 4j, Table 3:

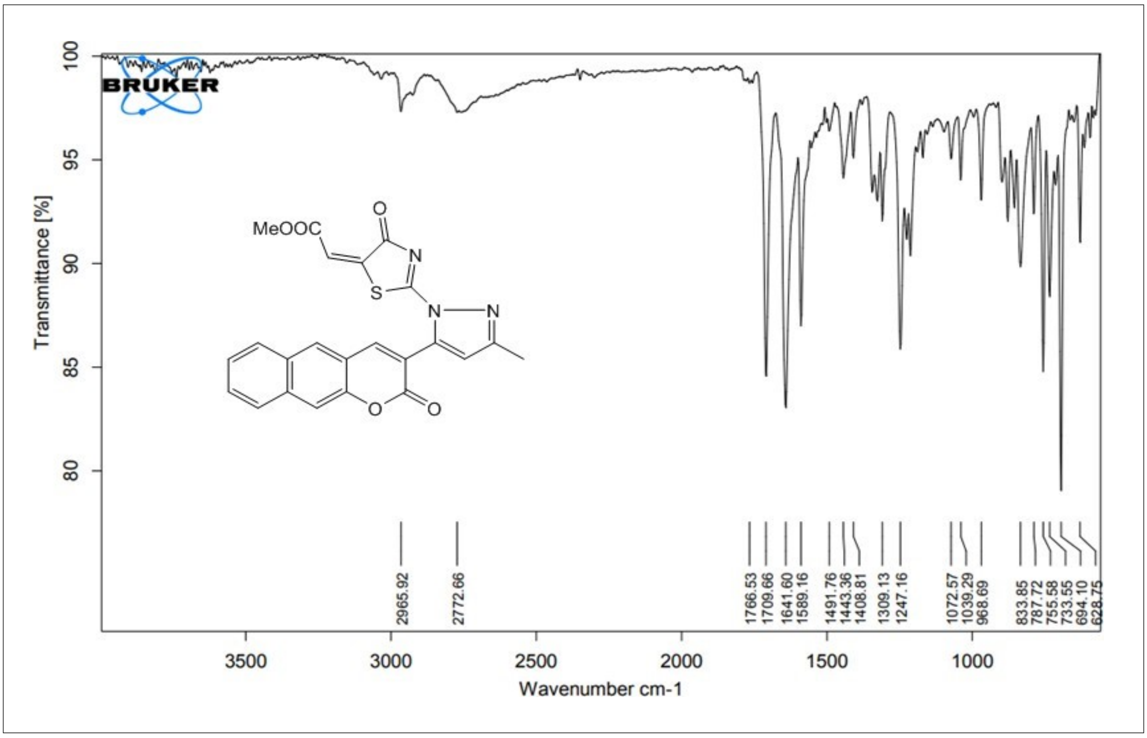

Fig. 37

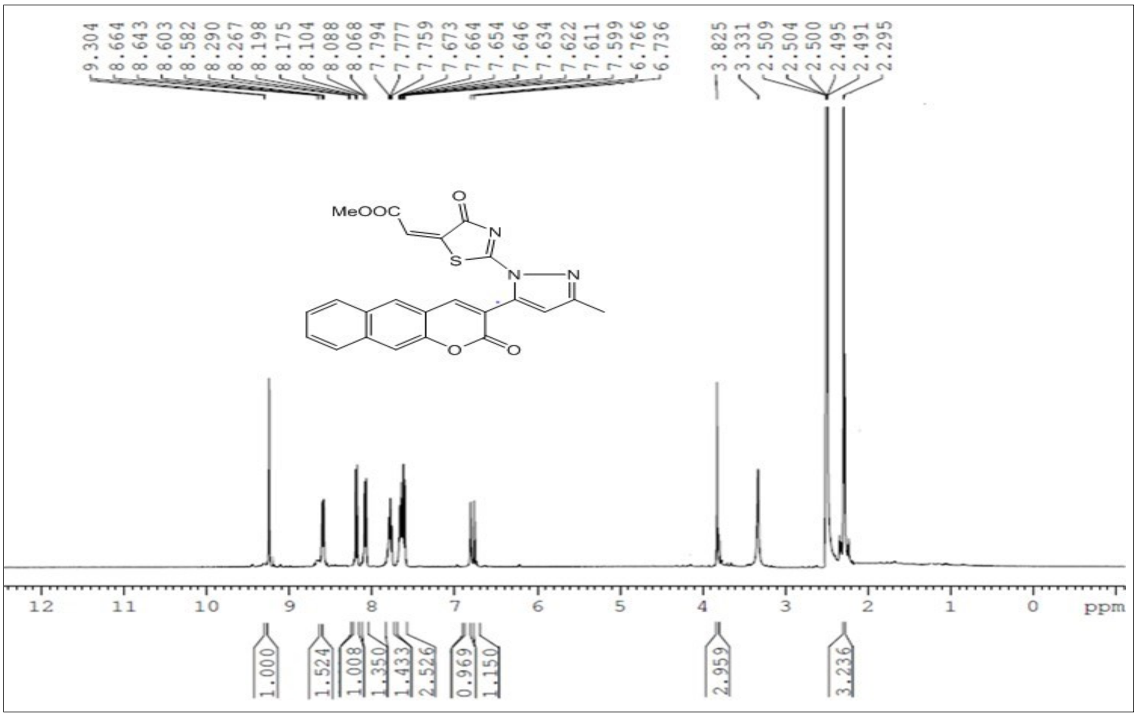
9.304
8.664
8.643
8.603
8.582
8.290
8.267
8.198
8.175
8.104
8.088
8.068
7.794
7.777
7.759
7.673
7.664
7.654
7.646
7.634
7.622
7.611
7.599
6.766
6.736
3.825
3.331
2.509
2.504
2.500
2.495
2.491
2.295
MeOOC
O
N
S
N
N
O
O
12 11 10 9 8 7 6 5 4 3 2 1 0 ppm
1.000
1.524
1.008
1.350
1.433
2.526
0.969
1.150
2.959
3.236

Fig. 38

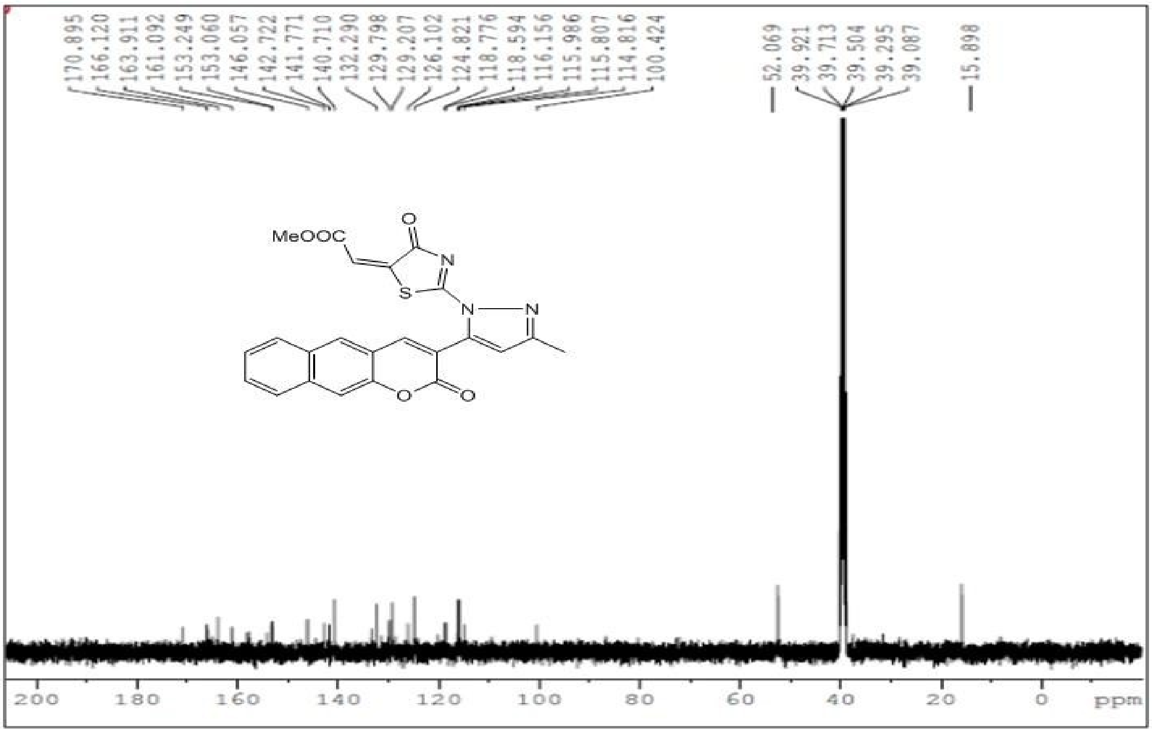
170.895
166.120
163.911
161.092
153.249
153.060
146.057
142.722
141.771
140.710
132.290
129.798
129.207
126.102
124.821
118.776
118.594
116.156
115.986
115.807
114.816
100.424
52.069
39.921
39.713
39.504
39.295
39.087
15.898
MeOOC
O
N
S
N
N
O
O
200 180 160 140 120 100 80 60 40 20 0 ppm

Fig. 39

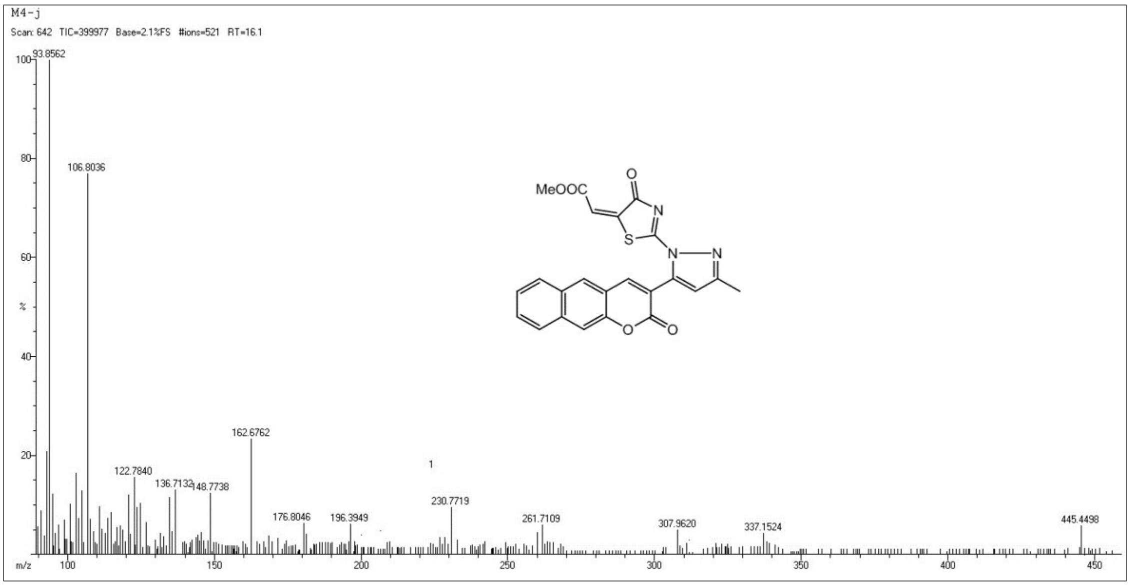
M4-j
Scan: 642 TIC=399977 Base=2.1%FS #ions=521 RT=16.1
93.8562
106.8036
122.7840
136.7132
148.7738
162.6762
176.8046
196.3949
230.7719
261.7109
307.9620
337.1524
445.4498
%
m/z
MeOOC
O
N
S
N
N
O
O

Fig. 40
